# The Unique Seed Protein Composition of Quality Protein Popcorn Promotes Growth of Beneficial Bacteria From the Human Gut Microbiome

**DOI:** 10.3389/fmicb.2022.921456

**Published:** 2022-07-14

**Authors:** Nate Korth, Leandra Parsons, Mallory J. Van Haute, Qinnan Yang, Preston Hurst, James C. Schnable, David R. Holding, Andrew K. Benson

**Affiliations:** ^1^Nebraska Food for Health Center, University of Nebraska–Lincoln, Lincoln, NE, United States; ^2^Department of Food Science and Technology, University of Nebraska–Lincoln, Lincoln, NE, United States; ^3^Department of Agronomy and Horticulture, University of Nebraska–Lincoln, Lincoln, NE, United States; ^4^Center for Plant Science Innovation–Beadle Center for Biotechnology, University of Nebraska–Lincoln, Lincoln, NE, United States

**Keywords:** gut microbiome, quality-protein popcorn, fructoselysine, lysine, butyrate, fermentation

## Abstract

The effects of fiber, complex carbohydrates, lipids, and small molecules from food matrices on the human gut microbiome have been increasingly studied. Much less is known about how dietary protein can influence the composition and function of the gut microbial community. Here, we used near-isogenic maize lines of conventional popcorn and quality-protein popcorn (QPP) to study the effects of the *opaque-2* mutation and associated quality-protein modifiers on the human gut microbiome. *Opaque-2* blocks the synthesis of major maize seed proteins (α-zeins), resulting in a compensatory synthesis of new seed proteins that are nutritionally beneficial with substantially higher levels of the essential amino acids lysine and tryptophan. We show that QPP lines stimulate greater amounts of butyrate production by human gut microbiomes in *in vitro* fermentation of popped and digested corn from parental and QPP hybrids. In human gut microbiomes derived from diverse individuals, bacterial taxa belonging to the butyrate-producing family *Lachnospiraceae*, including the genera *Coprococcus* and *Roseburia* were consistently increased when fermenting QPP vs. parental popcorn lines. We conducted molecular complementation to further demonstrate that lysine-enriched seed protein can stimulate growth and butyrate production by microbes through distinct pathways. Our data show that organisms such as *Coprococcus* can utilize lysine and that other gut microbes, such as *Roseburia* spp., instead, utilize fructoselysine produced during thermal processing (popping) of popcorn. Thus, the combination of seed composition in QPP and interaction of protein adducts with carbohydrates during thermal processing can stimulate the growth of health-promoting, butyrate-producing organisms in the human gut microbiome through multiple pathways.

## Introduction

Dietary nutrients and other dietary components produce direct effects on human cells, tissues, and organs. However, diet can also influence health outcomes by producing changes in the species composition and functions of the human gut microbiome (Ley et al., 2008). A number of studies have found associations between obesity, heart disease, and diabetes, with misconfigured taxonomic profiles and the functional composition of the gut microbiome (Bäckhed et al., 2004; Tang et al., 2013; Kostic et al., 2015). While many studies have examined how microbial communities can be perturbed through changes in diet (Turnbaugh et al., 2009; Wu et al., 2011; David et al., 2014), the dietary treatments in those studies are typically focused on fibers and/or lipids. Comparatively, little is known about the effects of seed protein on the human gut microbiome, and this gap is particularly relevant given the increasing interest in plant-derived protein sources for foods.

Most studies on microbiome utilization of food components compare different species of crop plants; however, substantial genetic and phenotypic diversity exists in individual crop species. Naturally occurring genetic diversity has and continues to enable the development of new varieties of existing crops with altered composition and, potentially, altered impacts on the human gut microbiome. Unlike shifts between cultivating different crop species, new varieties of existing crops can be more easily adopted and produced by existing farmers, and often avoid both the need to reformulate existing food products and cultural resistance to reformulating recipes used to make food at home. Quality-protein maize (QPM) is an example of a new crop variety with altered composition produced by plant breeders by selection on naturally occurring genetic variation (Babu et al., 2005; Manna et al., 2005).

Three grain crops, rice, wheat, and maize, are directly or indirectly responsible for half of all calories consumed by humans around the world (World Health Organization [WHO], 2003). While the composition of each of these grains is mainly composed of starch, grain protein can fulfill a significant proportion of total daily protein needs in both humans and animals (Li and Vasal, 2015). Unfortunately, the protein present in these grains is deficient in certain essential amino acids. Relative to human nutritional requirements, seed protein from maize is deficient in the essential amino acids lysine and tryptophan, which are not synthesized by the human body and must instead be obtained from dietary sources (Swendseid, 1981). Over 50% of the total protein in maize grain consists of a family of seed storage proteins called zeins, which are rich in the amino acids proline and methionine. The most abundant zein family, α-zeins, entirely lacks tryptophan residues and includes only minimal amounts of lysine. These unfortunate properties of zein proteins are the primary drivers of amino acid deficiencies in maize protein (Vasal et al., 1980; Sofi et al., 2009; Li and Vasal, 2015). Naturally occurring mutations in the *opaque-2* gene, encoding a transcription factor in maize, essentially abolishes the synthesis of α-zeins and triggers proteome rebalancing by increased synthesis of non-zein proteins into the seed (Mertz et al., 1964; Sofi et al., 2009; Li and Vasal, 2015). Although non-zein proteins from *opaque-2* mutants are not well-characterized, the total seed protein of *opaque-2* maize plants have significantly higher proportions of lysine and tryptophan (Mertz et al., 1964; Morton et al., 2016). The *opaque-2* mutation is pleiotropic but has additional phenotypic consequences beyond changes in seed protein composition. Homozygous *opaque-2* lines produce soft, chalky kernels that lack vitreousness (hard glassiness of the endosperm) and exhibit poor germination and increased susceptibility to many plant pathogens (Salamini et al., 1970; Bjarnason and Vasal, 2010).

To overcome undesirable phenotypes of *opaque-2* mutations, plant breeders have developed maize varieties that incorporate both the *opaque-2* mutation and naturally occurring allelic variants of unlinked modifier genes that ameliorate the negative agronomic properties of *opaque-2* while maintaining the desirable seed protein composition with higher lysine and tryptophan abundance (Vasal, 2000; Bjarnason and Vasal, 2010). These varieties are referred to as quality-protein maize (QPM) lines. In malnourished children, diets augmented with QPM have remarkably been shown to sustain growth rates equivalent to those of feeding with a modified cow milk formula (Graham et al., 1990). QPM has also been shown to promote health and disease tolerance (Akuamoa-Boateng, 2002), and height and body weight gains in older children (Akalu et al., 2010; Nuss and Tanumihardjo, 2011; Mamatha et al., 2017). Consequently, QPM is an outstanding illustration of how within-species genetic variation in food crops can have significant effects on human nutrition and wellness. Although these positive health attributes of diets with QPM compared to elite maize lines clearly establish the benefits of QPM in malnourished populations, there is no understanding of the involvement of the gut microbiome in mediating these health outcomes and, accordingly, whether such interactions could be beneficial in the populations on westernized diets.

Early QPM lines were developed in dent corn cultivars used for producing corn meal, industrial starch, and animal feed. However, the impact of dent corn varieties on human nutrition is largely manifested indirectly by feeding food animals or fractionation of the whole grain by food processing methodologies (industrial starch, corn syrup, etc.), which largely remove the protein component of the grain. In contrast, popcorn, which is genetically distinct from dent corn varieties, is directly consumed by humans as a whole grain around the globe. Thus, popcorn provides an excellent opportunity to study the relationship between quality protein characteristics, the gut microbiome, and human nutrition. Development of the quality-protein trait in cultivars of popcorn is challenging because of the need to introgress the *opaque-2* mutation along with unlinked modifier mutations from QPM dent lines into popcorn backgrounds to maintain both QPM protein phenotypes and phenotypes important for popping characteristics. This was recently accomplished in several different popcorn backgrounds, yielding quality-protein popcorn (QPP) lines that preserve both the quality protein and popping characteristics (Ren et al., 2018; Parsons et al., 2020, 2021a,b).

Here, we study the effects of seed protein composition on the human gut microbiome using a combination of popcorn varieties comprising pairs of non-quality-protein parental varieties and near-isogenic derivatives. The near-isogenic derivatives were generated by converting the same popcorn inbreds to a QPP by introgression of both *opaque-2* and quality-protein modifier genes (Ren et al., 2018; Parsons et al., 2021a). The relative impacts of protein composition in near-isogenic pairs of non-quality-protein parental hybrid (NQPH) and QPP derivatives on fermentation patterns of the human gut microbiome were studied in detail using batch *in vitro* fermentation reactions with stools of human donors. This approach is a powerful, preclinical strategy to evaluate the potential for diet-microbiome interactions. We found that overall microbial diversity, groups of beneficial species from the bacterial family *Lachnospiraceae*, and microbial production of butyrate are positively affected in fermentations where QPP was the substrate compared to NQPH.

## Materials and Methods

### Germplasm and Plant Growth Conditions

Three QPM lines (K0326Y, CML154Q, and Tx807) were employed as donor parents to introgress the *opaque-2* mutation and QPM modifiers into three existing elite popcorn inbreds from the ConAgra Brands^®^ popcorn breeding program (coded as P1, P2, and P3) (Ren et al., 2018). QPM popcorn lines were advanced to the BC2F5 generation by single seed descent (Parsons et al., 2020). K0326Y QPM was originally sourced from Gevers and Lake (1992), and CML154Q and Tx807 QPMs were obtained from the North Central Regional Plant Introduction Station. The F1 hybrid seed for QPP hybrids and respective non-QPM converted popcorn hybrids were generated in the spring of 2020 except for the P1 x P3 F1 seed, which was obtained from ConAgra Brands. Both QPM-converted popcorn hybrids and matched non-QPM-converted popcorn hybrids were grown in the summer of 2020 in a generalized complete block design in three locations with three replications. To prevent cross-pollination, all experimental units (four rows 17.5 feet in length) were separated by six rows of dent maize (incompatible pollination with popcorn) on all sides. The F1 seed was sown on 30 April 2020 in Lincoln, NE, United States (40°50′11.6″N 96°39′42.4″W DMS) and machine-harvested on 16 September 2020. Random samples from two randomly chosen EUs of the F2 harvested seed were used for analysis (Parsons et al., 2021a). The final QPP and NQPH nomenclature, parentage, and lysine content are reported in Table 1.

**TABLE 1:** Description of the pedigree of non-quality-protein parental hybrid (NQPH) lines and quality-protein popcorn (QPP) derivatives.

Quality protein popcorn			Non-QPM popcorn		
Nomenclature (Parsons et al., 2020)	Pedigree	Protein-bound lysine content (g/100 g)*	Respective hybrid nomenclature	Pedigree	Protein-bound lysine content (g/100 g)*
QPP1A (Hybrid 28)	{((CML154QxP1)xP1)F5} × {((K0326YxP2)xP2)F5}F2	0.241 ± 0.027	NQPH1	P1 × P2	0.120 ± 0.006
QPP1B (Hybrid 38)	{((CML154QxP1)xP1)F5} × {((K0326YxP2)xP2)F5}F2	0.325 ± 0.012	NQPH1	P1 × P2	0.120 ± 0.006
QPP2 (Hybrid 20)	{((K0326YxP2)xP2)F5} × {((CML154QxP1)xP1)F5}F2	0.221 + 0.013	NQPH2	P2 × P1	0.125 + 0.019
QPP3A (Hybrid 25)	{((CML154QxP1)xP1)F5} × {((CML154QxP3)xP3)F5}F2	0.164 ± 0.021	NQPH3	P1 × P3	0.133 + 0.007
QPP3B (Hybrid 43)	{((CML154QxP1)xP1)F5} × {((Tx807xP3)xP3)F5}F2	0.266 ± 0.035	NQPH3	P1 × P3	0.133 + 0.007

*Values as reported in Parsons et al., 2021a

### Microbiome Collection and Sample Processing

Stool samples from four adult volunteers with no GI disorders, antibiotic treatments, or probiotic supplementation in the previous 6 months were collected using a sterile commode specimen collection system. Dietary information for the subjects was not collected. The stool samples were diluted in PBS with 10% glycerol and homogenized using Interscience BagMixer 400 and filtered with the Labplas 6 × 9 filtra-bags. The filtrate was immediately frozen at −80°C for storage. The sample collection protocol was approved by The University of Nebraska-Lincoln’s Institutional Review Board (IRB 20160816311EP).

### Grain Sample Processing and Digestion

Grain samples from each hybrid were popped independently by line using a standard popping protocol in an Orville Redenbacher hot air popper without oil that simulates a common type of home and commercial popping that leads to the highest preservation of lysine content (Parsons et al., 2020). The popped flakes were flash frozen at –80°C and ground into a fine powder in Spex Geno/Grinder 2025 using one 7/16″ and three 5/16″ stainless steel ball bearings for 4 min at 1,500 rpm. The ground popcorn underwent *in vitro* digestion and dialysis to recreate the conditions of the upper human gastrointestinal tract. The digestion protocol that was adapted from established methods (Yang et al., 2013; Tuncil et al., 2018) was originally developed for carbohydrate research; therefore, we further modified it to achieve more complete digestion of starch and partial digestion of protein. To achieve this, we adjusted the amounts of α-amylase and pancreatin in the digestion protocol. In short, 2.5 g of sample was resuspended in 30 ml phosphate buffer and was treated with 0.8 U/ml amylase (Megazyme E-BSTAA), and then reduced to pH 2.5 ± 0.5 with 1M HCl and 0.1 mg/ml pepsin (Sigma P7000). The pH was raised to 6.9 ± 0.5 using 6 M sodium hydroxide, and the samples were then treated with 20 U/ml amyloglucosidase (Megazyme E-AMGDF) and 0.45 mg/ml pancreatin (Sigma P7545). This amount of pancreatin was considerably less proteolytic enzyme treatment than many standard protocols for digestion in carbohydrate-focused research (Yang et al., 2013; Chen et al., 2018) but is still in line with other previously reported digestion methods (Tuncil et al., 2018) and results in partial fragments of protein remaining in the digestate. To simulate the absorption of small molecules in the small intestine, the digested samples were placed in dialysis tubing (1,000 MWCO) and dialyzed against water for 72 h at 4°C.

Following digestion and dialysis, total protein and total starch were measured in each sample using Megazyme Total starch kit K-TSTA and a Bradford assay (He, 2011). Then, 0.45 ml of the digested material was aliquoted on 96-well plates and fermented for 16 h at 37°C under anaerobic conditions with 0.06 ml fecal samples from one of the four healthy individuals and 0.15 ml 4× fermentation media reduced at 4°C for 3 days. Fecal samples in media, before and after fermentation, we used as controls.

### 16S rRNA Sequencing and Quantification

The processing of the microbiome samples followed a previously published protocol (Zhu et al., 2020). In brief, DNA was extracted from cell pellets following fermentation using a BioSprint 96 One-For-All Vet kit (Qiagen). The V4 region of the 16S rRNA was amplified and indexed by PCR using primers described previously (Caporaso et al., 2010) (libraries were pooled, diluted, and QCd). Paired-end sequencing with 250 bp reads was performed on the Illumina MiSeq platform (Kozich et al., 2013) at the Nebraska Food for Health Center. Each sample was digested in two batches, and each batch was fermented in four independent wells of a 96-well plate for a total of eight replicates per sample per subject. Sequencing of 16S rRNA resulted in an average of 37,123 reads per sample (maximum 59,740, minimum 22,971).

### 16S rRNA Sequencing Data Processing

The DADA2 pipeline in QIIME2 (Callahan et al., 2016) was used to group exact amplicon sequence variants (ASVs) in each sample and assign taxonomy *via* the SILVA 16S database (Quast et al., 2013). Forward reads were trimmed to 220 bp, and reverse reads were trimmed to 160 bp to retain only high-quality sequence data. ASVs present in only one sample or composed of less than 15 reads were removed.

### Gas Chromatography

Short-chain fatty acids (SCFAs) were quantified by gas chromatography as described in detail by Yang and Rose (2014). In brief, aliquots of the supernatant from microbial fermentations were mixed with 0.1 ml 2-ethylbutyric acid to serve as an internal standard, followed by salination, acidification, and extraction into diethyl ether. One μl of diethyl ether was injected onto a gas chromatograph (Agilent 8890 GC system) with a 15 m × 0.25 mm × 0.025 μm capillary column and detected with a flame ionization detector. SCFAs were quantified by calculating the area under each known SCFA peak relative to the internal standard using injections of a standard solution (Yang and Rose, 2014).

### Lysine and Fructoselysine Validation

To test the hypothesis that the *Lachnospiraceae* and butyrate enrichment observed *in vitro* in response to QPP was due to higher contents of lysine and/or fructoselysine, the microbiome of S1 was used in a simple medium with the addition of lysine or fructoselysine adapted from published protocols (Bui et al., 2015). In short, the bicarbonate buffer medium adapted from Stams et al. (1993) contained 0.41 g/L K_2_HPO_4_, 4 g/L NaHCO_3_, 0.3 g/L NaCl, 0.53 g/L Na_2_HPO_4_, 0.3 g/L NH_4_Cl, 0.11 g/L CaCl, 0.1 g/L MgCl_2_, 0.48 g/L Na_2_S, 0.6 ml/L resazurin, 10 ml/L ATCC trace minerals, and 10 ml/L ATCC vitamin supplement. Microbial fermentations were completed for 16 or 32 h in media alone or media containing 20 mM L-lysine (Sigma-Aldrich L5501), or 10 mM a-Fructoselysine (US-Biological 167175); 16S rRNA sequencing and SCFA quantification were performed as detailed in the previous sections.

### *Roseburia* Species Analysis

To accurately measure the growth of *Roseburia* at a species level in response to lysine and FL, a previously reported qPCR protocol with primers for four species was employed (Yang et al., 2022). In brief, species-specific primers were designed with the Rapid Identification of PCR Primers for Unique Core Sequences (RUCS) software (Thomsen et al., 2017) and validated with Primer BLAST against the RefSeq representative genome database (Ye et al., 2012). The primer sequences outlined in Table 2 were synthesized by Integrated DNA Technologies (Coralville, IA, United States). To approximate the CFU/ml values for each species, the cycle threshold was compared to a standard curve generated by diluting DNA from *Roseburia intestinalis* (DSM 14610), *Roseburia inulinivorans* (DSM 16841), *Roseburia hominis* (DSM 16839), and *Roseburia faecis* (DSM 16840).

**TABLE 2:** Primers used in qPCR quantification of *Roseburia* species.

Species	Forward primer	Reverse primer
*R. intestinalis*	CGAAGCACTTTATTTGATTTCTTCGG	TTTTTCACACCAGGTCATGCG
*R. hominis*	AAGTCTTGACATCCCACTGACA	CACCACTGCTCCGAAGAGAA
*R. inulinivorans*	GACATCCTTCTGACCGGACAG	GGCTACTGGGGATAAGGGTTG
*R. faecis*	CGCAACCCCTGTCCTTAGTAG	AGATTTGCTCGGCCTCACG

### Statistical Analyses

Alpha and beta-diversity analyses and visualization were conducted using ATIMA (Alkek Center for Metagenomics and Microbiome Research). R statistical software v4.0.2 with the Sommer package was used to perform a statistical analysis including linear mixed modeling calculations to compare variation in single ASVs explained by QPP and total starch and protein ratio (Covarrubias-Pazaran, 2016; R Core Team, 2020). Plots were made using ggplot2. The phylogeny inferred by QIIME2 was plotted in iTree of Life (Letunic and Bork, 2021). PICRUSt2 was employed to predict the metagenomic composition of microbiome samples based on ASVs (Douglas et al., 2020).

## Results

### Pre-fermentation Analysis of Fecal Microbiomes and Popcorn Composition

The effects of QPP and NQPH were assessed in microbiomes sourced from stool samples of the four different human donors with diverse microbiome compositions. Inclusion criteria for the subjects included an absence of known gastrointestinal diseases and no history of antibiotic or probiotic consumption in the past 6 months. Diversity in the taxonomic composition of the baseline microbiomes from the four human subjects is illustrated at the genus level (Supplementary Figure 1), where abundances of common gut microbial taxa such as *Bacteroides* (9.2–20.9%), *Prevotella* (0–28%), *Agathobacter* (1.5–9.5%), *Anaerostipes* (2.5–9%), *Roseburia* (0–4.1%), *Blautia* (6.7–14%), and *Phascolarctobacterium* (0–4.5%) vary considerably between subjects.

Previously, a set of NQPH parental popcorn lines and multiple QPP derivatives from each parental line were successfully generated and characterized (Parsons et al., 2020, 2021a). The same sets of NQPH parental and QPP lines were used for our experiments.

We first evaluated major seed composition profiles from NQPH and QPP derivatives with respect to total starch and protein content post-digestion (Supplementary Figure 2). Total protein content from the modified digestions was significantly higher in the QPP (0.97%) than in the NQPH lines (0.83%) (Wilcoxon *p* = 2.75e-05) and significant differences between individual pairs of NQPH parental lines and QPP derivatives. The residual starch content of QPP hybrids was significantly lower than that of NQPH (means of 6.5% starch in QPP vs. means of 7.7% starch in NQPH; Wilcoxon *p* = 3.14e-06), reflecting the known pleiotropic effect of the *opaque-2* mutation in reducing starch content of the kernel (Gibbon et al., 2003). However, there were no significant differences in starch content between individual QPP hybrids or between individual NQPH.

### Microbiome Analysis of Fermentations With QPP and Non-quality-Protein Parental Hybrid Lines

For *in vitro* fermentation, digested and dialyzed components from the popped seed of the QPP and NQPH lines were used as substrates and introduced to microbiomes from the four different human subjects (S1–S4), and the reactions were incubated anaerobically at 37°C. Amplicon sequencing of the V4 region of the rRNA gene was subsequently performed on samples before and after fermentation, including controls in which no popcorn was introduced. The microbiome data were evaluated for different metrics of overall ecological diversity, abundances of individual microbial taxa, and primary microbial fermentation metabolites.

**FIGURE 1:**
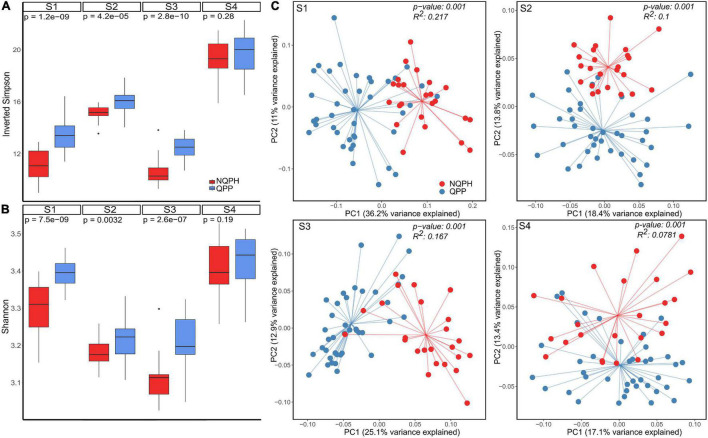
**(A)** Diversity reported as Inverted Simpson and **(B)** Shannon metrics of α-diversity for global differences between the microbial response to quality-protein popcorn (QPP) and non-quality-protein parental hybrid (NQPH); *p*-values were calculated by Wilcoxon test. **(C)** Jaccard index of β-diversity coupled with PCoA ordination shows total differences between microbiome response to QPP and NQPH.

As shown in Figure 1, α-diversity was elevated in fermentations of QPP lines vs. NQPH lines in three of the four subjects, and this difference was statistically significant (FDR-adjusted Wilcoxon test). The most abundant taxa across the reactions (Supplementary Figure 3) were consistent with the QPP vs. NQPH line effects on α-diversity, driven by relatively small but measurable shifts in the relative abundance of multiple taxa that affect the evenness and richness of the taxa. Across the three subjects with significant differences in α-diversity, 13 of 20 (inverted Simpson index) and 12 of 20 (Shannon index) fermentations of individual QPP hybrids exhibited significantly higher diversity than their NQPH counterparts, with most of the significant changes observed in microbiomes of subjects S1–S3 (Figure 1A and Supplementary Figure 4). All four subjects showed statistically significant differences in the overall taxonomic configuration of the microbiome between the NQPH lines and the QPP lines as quantified by β-diversity, which measures between-treatment diversity (Jaccard distance tested for significance by PERMANOVA, *p* < 0.01) (Figure 1B). Both α- and β-diversity metrics point toward significant effects of the unique protein composition of the QPP and NQPH lines on overall microbiome composition, and the differences largely manifest as a combination of abundance changes in several different taxa. Notably, two of the QPP hybrids, QPP1B and QPP3B, did not frequently exhibit significant differences in microbial diversity relative to their paired parental hybrid lines (Supplementary Figure 4).

Statistically significant differences in abundances of a number of individual taxa were also detected in the fermentation of QPP vs. NQPH lines. Statistically significant differences in abundances in the fermentation of popcorn from QPP vs. NQPH lines (Mann–Whitney, *p* < 0.05) were observed between microbial taxa at different levels of the taxonomic hierarchy (e.g., family, genus, etc.) evaluating only the taxa present at a level of at least 0.1% relative abundance per subject. Individual amplicon sequence variants (ASVs), the most specific level of taxonomy identifiable by sequencing the V4 region of the 16S rRNA gene (Supplementary Table 1), are related to different taxonomic units in a genus but are often difficult to attribute to individual species. Ninety-four ASVs present in one of the four subjects showed statistically significant differences in abundances in fermentations from QPP vs. NQPH popcorn, including some that overlapped in at least three of the subjects (*Eubacterium*-ASV28, *Anaerostipes*-ASV30, *Bacteroides*-ASV17, *Blautia*-ASV20, *Blautia-*ASV7, and *Coprococcus*-ASV15) (Supplementary Table 2). In some cases, such as the genus *Bacteroides*, individual ASVs assigned to the taxon increased in abundance while other ASVs assigned to the same taxon decreased, producing signals observable at the individual ASV level that were not captured when ASVs were aggregated into higher level taxonomic groupings.

**FIGURE 2:**
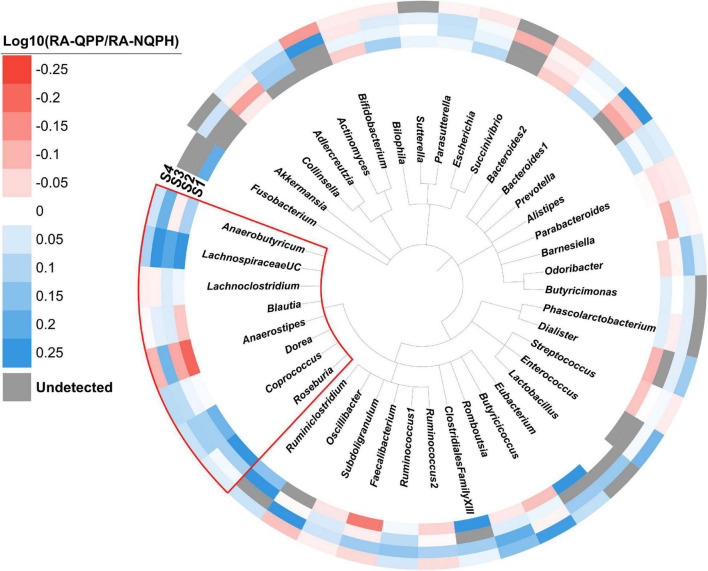
Phylogeny of the genera observed in the four subjects. The colored ring denotes the log ratio of the relative abundance of each genus in microbiomes treated with QPP to NQPH. Gray bars indicate the genus was not present at a detectable level. The red box indicates members of the *Lachnospiraceae* family.

When ASVs were aggregated at the genus and/or family levels, statistically significant responses to QPP vs. NQPH treatment were observed for fifty-five genera that corresponded to twenty-four families (Supplementary Table 2). At the genus level, 23 of the 55 genera showing statistically significant responses in at least one subject were significant in at least two subjects, and one genus (*Coprococcus* 3) was significant in three. Overall, the patterns of organisms that showed significant increases or decreases in abundance in fermentations of QPP vs. NQPH was highly individualized to each microbiome (Figure 2). Even at the genus level, many taxa displayed differential responses in each subject, implying some dependence on the overall microbiome context. For example, organisms such as *Faecalibacterium* and *Anaerostipes* were significantly less abundant in microbiomes treated with QPP across subjects 1, 2, and 4 but significantly higher in subject 3. Even with the strong effect of the microbiome context on taxonomic responses, a small number of organisms had common patterns between two or more subjects. For example, *Bacteroides 2* was consistently less abundant in microbiomes treated with QPP while taxa, many belonging to the *Lachnospiraceae*, were often significantly more abundant in microbiomes treated with QPP.

Perhaps one of the most striking findings with respect to the overall taxonomic responses of the microbiomes was the correlated behavior of many taxa belonging to the family *Lachnospiraceae* (phylum Firmicutes), which were generally significantly more abundant in the fermentations with QPP lines in two or more individual microbiomes (Figure 2 and Supplementary Figure 5). *Coprococcus* showed a consistent response across the four microbiomes, with the aggregate abundance of ASVs belonging to the genus *Coprococcus* also being significantly higher in three or more QPP hybrids than their cognate NQPH lines in three of the subjects (Supplementary Figure 6). The abundance of ASVs belonging to *Roseburia* and *Dorea* and aggregate abundance of all ASVs assigned to these genera were also elevated in microbiomes treated with QPP relative to NQPH in two of the four microbiomes. These organisms are generally considered to be saccharolytic, and their ability to utilize dietary peptides/amino acids has not been well studied. However, because the *opaque-2* mutation reduces resistant starch content in the QPP lines vs. the NQPH lines (Supplementary Figure 2), the elevated abundance of these organisms in QPP lines supports the conclusion that these organisms may be utilizing protein or other non-starch substrates.

### Microbiome Changes Are Associated With Changes in Short-Chain Fatty Acid Production

**FIGURE 3:**
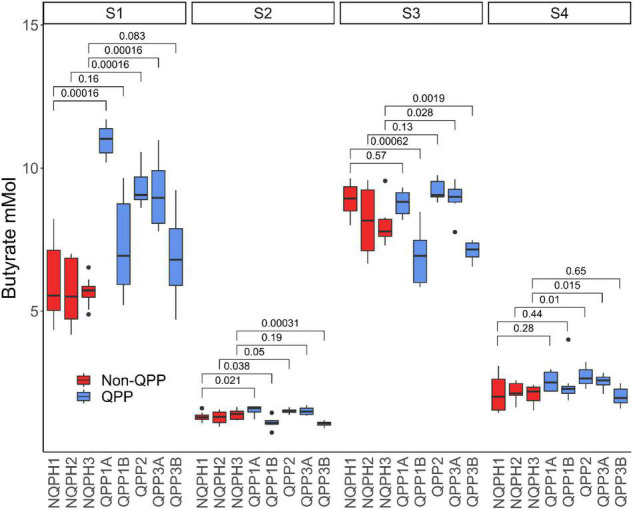
Butyrate concentration in microbiomes of the four subjects post treatment with QPP and NQPH lines and comparisons (Wilcoxon) between hybrid pairs. Red boxes denote hybrid pairs where the microbiome treated with a QPP hybrid has significantly less butyrate than the NQPH control.

Members of the family *Lachnospiraceae* found in human gut microbiomes are recognized for their ability to produce butyrate as a primary product of carbohydrate fermentation (Meehan and Beiko, 2014). Butyrate produces a number of beneficial effects on the gastrointestinal tract (Hague et al., 1996; Velázquez et al., 1997; Zhou et al., 2006; Wang et al., 2012; Shang et al., 2016). To determine if the observed differences in the microbiome by the QPP lines lead to corresponding differences in butyrate production (e.g., from amino acid degradation), we first measured short-chain fatty acids from the *in vitro* fermentation reactions (see Materials and methods). Here, we identified consistent and statistically significant differences in butyrate concentration from the fermentation of QPP vs. NQPH substrates in two donor microbiomes (Supplementary Figure 7). As with the differences in microbial abundance, the patterns of difference in butyrate production between the QPP and NQPH lines were unique to each individual microbiome. Subject 1 exhibited significantly higher butyrate in all QPP lines relative to NQPH controls. In the other subjects, only two QPP lines showed significantly more butyrate than their cognate NQPH. Nonetheless, in each subject, at least one QPP line stimulated a significantly greater abundance of butyrate than the cognate NQPH parental line (Figure 3).

Significant changes in the production of acetate and propionate were also observed. In general, the abundance of both acetate and propionate was negatively correlated with the abundance of butyrate (Supplementary Figure 8A). In reactions from QPP lines where butyrate production was elevated relative to the cognate NQPH line, the abundances of acetate and propionate were significantly reduced. This pattern was particularly apparent for the microbial communities from subjects 1 and 2, where fermentation of QPP lines favored the production of butyrate, whereas fermentation of NQPH hybrids favored the production of propionate and acetate. The branched chain fatty acids iso-butyrate and iso-valerate, which are generally derived from the fermentation of proteins, were also quantified (Supplementary Figure 8B). Iso-valerate was significantly higher in NQPH than in QPP lines in subjects 3 (Wilcoxon *p* = 2.8e-05) and 4 (Wilcoxon *p* = 1.9e-3). This is expected, since leucine content, the primary substrate for fermentation to iso-valerate, is substantially lower in QPP lines. Similarly, valine is slightly lower in QPP lines, and its branched chain fatty acid product, isobutyrate, was higher in NQPH than in QPP in subjects 2 (Wilcoxon *p* = 4.3e-2), 3 (Wilcoxon *p* = 3.3e-03), and 4 (Wilcoxon *p* = 2.3e-2).

**FIGURE 4:**
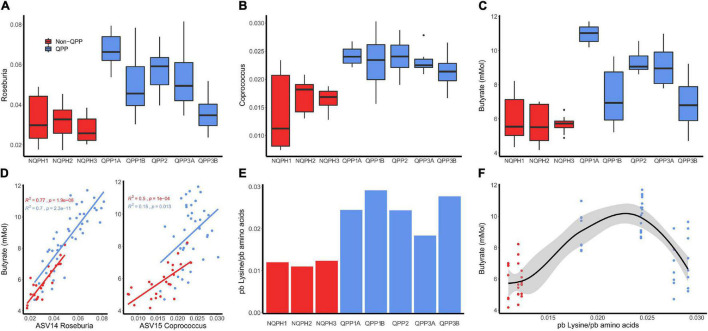
Relative abundances of **(A)** *Roseburia* and **(B)** *Coprococcus* in the microbiome of subject 1 after treatment of QPP and NQPH pairs. Butyrate concentration was highly correlated with the relative abundance of **(C)** ASV13 *Roseburia* and, to a lesser extent and ASV15 *Coprococcus* **(D)**. The measured amount of pre-digestion, protein bound lysine **(E)** is linearly correlated to butyrate concentration **(F)**.

**FIGURE 5:**
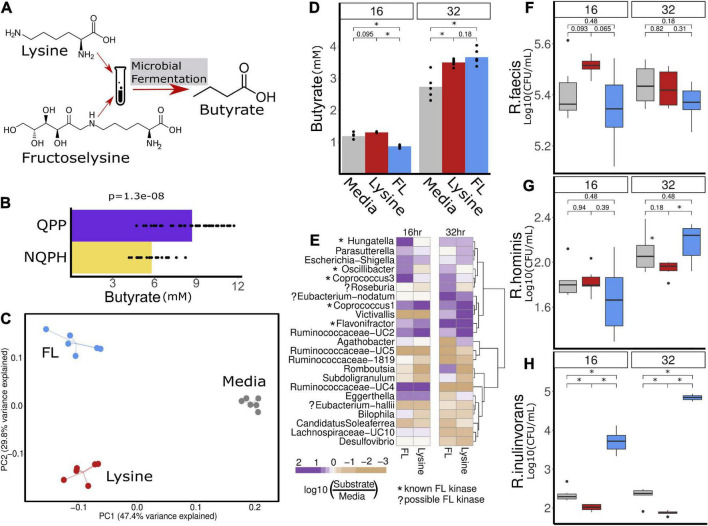
**(B)** Hypothesized mechanism of action for the stimulation of butyrate production in the microbiome of S1 in response to QPP is the conversion of lysine and/or FL to butyrate. **(C)** Community structure measured as β-diversity (Jaccard index) indicates differences in microbial composition when treated with lysine or FL. The significant increase in butyrate production in the microbiome of S1 in response to QPP compared to NQPH **(A)** was recapitulated in treatment with lysine and FL in 32-h fermentation **(D)**. Multiple organisms were identified by 16S sequencing to be enriched by lysine and/or FL, many of which contain known genes implicated in the conversion of FL to butyrate **(E)**. Species level investigation of *Roseburia* by qPCR identified no response of **(F)** *R. faecis* or **(G)** *R. hominis* to FL or lysine, while the content of **(H)** *R. inulinivorans* was significantly increased at both 16 and 32 h in response to treatment with FL. *denotes statistical significance of *P*-value calculated by the Wilcoxon rank-sum test where alpha < 0.05.

The increase in butyrate production in response to QPP in S1 was considerably larger than the increases observed in the other subjects (Figures 3, 4C, and 5B). The correlation analysis showed a significant association of the abundances of Roseburia and *Coprococcus* with butyrate concentration in both QPP and NQPH lines in this individual microbiome (Figures 4A–D), suggesting these organisms are potential contributors to the pool of butyrate produced during fermentations. The relationship between propionate and acetate and other taxa varied widely by subject. Both acetate and propionate were correlated with each other (S1, S3, and S4), total starch content (S1 and S2 only propionate; S3 and S4 only acetate), and a number of bacterial ASVs including prevalent ASVs of *Bacteroides* (S1–S4), *Blautia* (S1 and S2 only acetate) and *Faecalibacterium* (S1).

### Lysine and Fructoselysine Can Serve as Fermentation Substrates for the Microbiome

Quality-protein popcorn lines have significantly higher lysine content than NQPH lines (Parsons et al., 2021a; Figure 4E), and these lines stimulate higher levels of butyrate production during fermentation (Figure 3). One potential mechanism to explain these observations is that microorganisms stimulated by QPP lines that drive high levels of butyrate production would have pathways for the degradation of lysine to butyrate. A PICRUSt (Douglas et al., 2020)-based analysis to infer functional pathways from 16S data was conducted as a strategy to generate hypotheses explaining these unexpected results. Starch degradation pathways were enriched in the microbiomes from the fermentation of NQPH lines in the PICRUSt analysis of the fermentations of subject 1, the subject that displayed the highest level of taxonomic and metabolic (butyrate) responsiveness to QPP vs. NQPH lines (Supplementary Figure 2). In contrast, significant enrichment of both the acetyl-Co-A pathway for butyrate production and the primary pathway for L-lysine fermentation to butyrate was observed in the QPP lines (Supplementary Figure 10). PICRUSt identified ASVs belonging to *Coprococcus, Roseburia, Blautia, Bacteroides, and Fusobacterium* as the most likely to carry genes in the L-lysine fermentation pathway to acetate and butyrate (BiocycID: P163-PWY). However, studies on metagenomes from *Lachnospiraceae* have shown that these organisms generally lack the 3-aminobutyryl-CoA pathway for lysine fermentation to butyrate (Vital et al., 2014). A closer examination of representative genomes from members of the *Lachnospiraceae* by BLAST analysis confirmed that these organisms typically carry enzymes for the acetoacetate pathway that enables fermentation of carbohydrates to butyrate, but that many of the representative species do not carry the genes for the 3-aminobutyryl-CoA lysine fermentation pathway. Thus, this pathway alone would not explain the correlation between elevated taxonomic abundances and elevated butyrate production in the fermentation of QPP vs. NQPH lines.

Species of *Intestinimonas* that have been studied in detail carry the genetic and enzymatic capacity for butyrate production from fermentation of lysine through the 3-aminobutyryl-Co-A pathway as well as production of butyrate from the substrate fructoselysine (FL) through an independent pathway (Bui et al., 2015). FL is produced from lysine by the Maillard reaction, where heating of reducing sugars in the presence of free amines (e.g., the gamma-amino group of lysine) leads to an Amadori reaction linking the nitrogen in the free amine to reducing sugars, which produce fructoselysine (Hellwig and Henle, 2014). Given the elevated levels of lysine residues in QPP seed protein and the high temperatures achieved during popping, FL is very likely to be produced at biologically relevant levels during popping. Thus, a plausible hypothesis is a thermal process of popping kernels from QPP lines results in much higher levels of FL in QPP lines than NQPH controls and FL could be driving the abundance increases and butyrate production of some members of *Lachnospiraceae* in QPP lines. A sensory analysis of the QPP and NQPH lines revealed “nutty” and “sweet” flavors more present in many of the QPP than the NQPH lines. The differential flavor profile supports the hypothesis that the Maillard reaction results in novel compounds including antioxidants, pyrazines, and pyrroles (Parsons et al., 2021b). Although FL content has not been reported for popcorn or QPP, estimates of FL content in baked goods suggest that as much as 1.5 mmol may be formed in 500 g of a baked good (Henle, 2003).

Using an established amino acid sequence for the FL kinase present in *Intestinimonas* (Bui et al., 2015), a BLAST analysis identified putative orthologs (>50% identity across >90% of alignment) in representative genomes of multiple taxa belonging to *Lachnospiraceae*, including *Coprococcus catus*, *Enterocloster asparagiformis*, *Anaerotignum lactatifermentans*, *Dorea longicatena*, *Blautia faecicola* and a single isolate of *Roseburia faecis*.

Based on the genome analyses, we developed an experimental strategy to differentiate whether lysine and/or FL were able to stimulate changes in taxonomic abundances and butyrate production that reflect our results with the QPP and NQPH lines. Using the S1 microbiome and a simple bicarbonate buffer medium, we introduced pure lysine or FL as the primary carbon substrate and quantified changes in the microbiome as well as butyrate production (Figure 5A). The addition of lysine or FL to fermentations resulted in substrate-specific changes in microbial community structure as indicated by β-diversity (Figure 5C). After a 32-h fermentation, the levels of butyrate in samples treated with lysine or FL were also significantly higher than in the medium with no substrate (Figure 5D). The abundances of several genera showed significant substrate-specific differences in relative abundances of taxa in fermentation (Figure 5E). With respect to lysine fermentation, *Hungatella, Parasutterella, Escherichia/Shigella, Oscillibacter, Coprococcus 1, Eubacterium nodatum group, Victivallis*, and *Ruminococcaceae-UC2, Ruminococcaceae-UC4* and *Eggerthella* all increased in abundance at either 16 and/or 32 h of fermentation. However, when FL was added as a substrate, *Hungatella, Parasutterella, Escherichia/Shigella, Oscillibacter, Coprococcus 3*, and *Ruminococcaceae-UC4* appeared to show preferential growth on FL at 16 and 32 h, whereas the *Eubacterium nodatum* group, *Flavonifractor*, and *Roseburia* showed preferential abundance changes with FL but not lysine.

Given the high degree of correlation between *Roseburia* and butyrate production (Figure 4D), the significant increase in abundance of *Roseburia* in fermentations supplemented on FL at 32 h and the presence of the FL kinase in a known isolate of at least one species (*Roseburia faecis*), we conducted species-specific qPCR to quantify the absolute levels of the four main *Roseburia* species and determine if abundance increases in the 16S data correspond to apparent growth of one or more species on the FL substrate. A single species, *Roseburia inulinivorans*, in S1 appeared to grow readily on the FL substrate and likely accounted for the increased abundance of *Roseburia* in the 16S rRNA data from the same fermentation (Figures 5F–H). Compared to the control medium or medium + lysine, the levels of *R. inulinivorans* in the medium supplemented with FL increased by >1 log at 16 h and nearly 2.5 logs after 32 h of fermentation (Figures 5F–H). Thus, certain *Roseburia* species appear to have the potential for selective growth on FL, even in the context of an intact microbiome. The capacity of the population of *R. inulinivorans*, but not *R. faecis*, in the S1 microbiome to grow on FL illustrates that the capacity for growth of *Roseburia* species on FL is likely to be strain-specific and that the presence of the pathway for FL degradation is not necessarily well-represented among available reference genomes.

## Discussion

With the dramatically increasing size of the global human population, there is increasing interest in the use of plant-derived protein as a sustainable means to offset the reliance on animal-derived protein in human diets (Kingston-Smith et al., 2010; Willett et al., 2019). While many plant-derived sources of protein are being explored, there has been little systematic effort to understand the influences of plant-derived protein sources on the human gut microbiome. In this exploratory study, we have begun to fill this major research gap by testing the hypothesis that major differences in seed protein composition can have distinct effects on the human gut microbiome.

Our experimental approach exploited *in vitro* fermentation with human stool microbiomes as a cost-effective approach for detailed pre-clinical evaluation of the capacity of specific dietary substrates to influence the human gut microbiome, bypassing costly human feeding studies (Mayta-Apaza et al., 2018). We coupled this robust approach to pre-clinical microbiome phenotyping with a powerful genetic strategy for perturbing seed protein composition in otherwise near-isogenic lines. The collective strategy allowed us to compare the effects of variation in protein content from near-isogenic pairs of NQPH and QPP lines in the context of whole grain, as opposed to biochemical approaches where purified components (e.g., purified seed protein from QPP vs. NQPH lines) are studied independently of the whole grain matrix. We suggest that this approach is particularly relevant for foods such as popcorn that are consumed as whole grain.

The combined phenotypes of the *opaque-2* mutation and alleles at modifier loci in QPM donor lines cause major proteome rebalancing in the seed, replacing α-zeins with proteins that have a much higher content of the essential amino acids lysine and tryptophan. Seed protein phenotypes, with respect to lysine and tryptophan content in the donor QPM lines and the QPP derivatives we studied (which carry *opaque-2* and modifier alleles from QPM donor lines), are very similar (Ren et al., 2018; Parsons et al., 2021a). Health benefits of the enhanced essential amino acid content of seed protein in QPM have been demonstrated in a number of human feeding studies, including increased growth rates, height, and body weight gains in malnourished children (Graham et al., 1990; Akalu et al., 2010; Nuss and Tanumihardjo, 2011; Mamatha et al., 2017), and disease tolerance in older children (Akuamoa-Boateng, 2002). Thus, the quality-protein phenotype has a significant potential for nutritional improvement, and foods enriched in quality proteins from QPM or QPP could have a wide range of potential health benefits.

We observed significant effects of the quality-protein phenotype in maize on the human gut microbiome. Although our study focused on a pre-clinical *in vitro* fermentation model, we suggest that our results support the possibility that quality protein in maize can elicit human health benefits through nutrient-host interactions as well as nutrient-microbiome-host interactions. Indeed, our results beg the question of whether improvement in growth rates, height, and body weight gains observed in malnourished children consuming QPM (Akalu et al., 2010; Mamatha et al., 2017) are manifested solely through nutrient-host interaction or if they also include beneficial effects on the host microbiome.

The detailed analysis of the effects of near-isogenic QPP vs. NQPH lines on *in vitro* microbiome fermentations revealed several statistically significant effects of QPP on the human gut microbiome. The effects could be observed on the overall composition of microbiomes (α- and β-diversity) from diverse microbiomes of the four different subjects (Figure 2), and similar effects were detected in SCFA metabolites such as butyrate (Figure 3 and Supplementary Figure 7). The abundance of multiple bacterial taxa was also altered between fermentations of QPP and NQPH popcorn. The combinations of taxa showing significant differences were largely microbiome-specific, with the exception of the QPP-driven effect of increasing the abundances of butyrate-producing members of *Lachnospiraceae*, which was shared across fermentations with microbiomes of each of the four subjects (Figure 2 and Supplementary Figure 4). Many members of *Lachnospiraceae* are considered beneficial microorganisms in the human gut microbiome, as their abundances have been shown to be correlated with reduced susceptibility to metabolic or inflammatory diseases in multiple studies (Meehan and Beiko, 2014). The ability of the novel seed protein from QPP to stimulate members of *Lachnospiraceae* across diverse human microbiomes in our study suggests that the composition of quality seed proteins may constitute a mechanism to specifically modulate this group of beneficial microbes across diverse human microbiome configurations.

Further evidence of the capacity of QPP lines to modulate members of *Lachnospiraceae* was obtained from the corresponding effects of QPP lines on the production of butyrate, the primary fermentation product of many members of *Lachnospiraceae*. Abundances of *Coprococcus* and *Roseburia* (both members of *Lachnospiraceae*) are highly correlated with butyrate concentration in the fermentation reactions in the most responsive microbiome (from subject 1) (Figure 4). The positive association of *Lachnospiraceae* with reduced disease susceptibility is believed to be mediated, in part, by the production of butyrate, which has a number of beneficial effects on the colon and extra-intestinal tissues. Butyrate is known to serve as a primary energy source for colonocytes (Hague et al., 1996), control the formation of tight junctions (Wang et al., 2012), influence the production of satiety hormones (Zhou et al., 2006), and control colonic motility (Velázquez et al., 1997). Butyrate is also known to reduce the production of inflammatory mediators and reduce lipid deposition in the liver (Shang et al., 2016).

*Lachnospiraceae* are widely known for their metabolic capacity to produce butyrate as a primary end product of carbohydrate fermentation through the acetoacetate pathway (Meehan and Beiko, 2014). Production of butyrate from lysine, however, funnels through a separate pathway (3-aminobutyryl-CoA pathway), but this pathway is generally absent in the genomes of many members of *Lachnospiraceae* (Vital et al., 2014). This pathway is, however, present in other gut-associated species belonging to Eubacteriales, Actinobacteria, Fusobacteria, and some members of Bacteroidetes, some of which were significantly more abundant in fermentations of QPP lines. Thus, the ability of QPP to enhance abundances of *Lachnospiraceae* and drive correlated increases in butyrate in our study was somewhat enigmatic. We investigated these puzzling results through a combination of comparative genomic analysis and growth experiments with purified substrates. Comparative genomics corroborated the absence of 3-aminobutyryl-CoA pathways in representative genomes of species from *Lachnospiraceae* but also provided evidence for the presence of pathways for conversion of FL to butyrate. FL is produced through the Maillard reaction where Amadori products from covalent crosslinking of free gamma-amino groups in protein are produced in the presence of reducing sugars at high temperatures (Namiki and Hayashi, 1983). Although FL content has not been reported for popcorn or QPP, given the high temperatures used for popping and the high lysine content of QPP, we would expect FL to be produced during popping of QPP popcorn.

When we tested this hypothesis by the addition of pure lysine or FL to fermentation of the S1 microbiome, there was clear evidence for the capacity of each substrate to differentially drive significant changes in the abundances of multiple taxa, including *Coprococcus* and *Roseburia* from the *Lachnospiraceae* family. A deeper exploration of the lysine and FL-driven responses of the four primary species of *Roseburia* by qPCR provided clear evidence that FL can indeed selectively stimulate the growth of *R. inulinivorans*, accounting for the FL-induced changes in 16S data from the same fermentation.

Clearly, much work remains to determine the precise mechanisms through which quality protein can influence the microbiome. Although we have focused on the benefits of the high lysine content of QPP on the microbiome, it remains to be determined whether any harmful products of protein metabolism (e.g., phenols, p-cresol, and ammonia) are elevated in the fermentation of QPP vs. NQPH lines (Nyangale et al., 2012). Our study lays the groundwork to justify experiments on pre-clinical animal models (e.g., gnotobiotic mice carrying human microbiomes) and human feeding studies to determine if quality protein (QPM and QPP) can influence the microbiome *in vivo* and if dietary protein-microbiome interactions may indeed be associated with health benefits.

## Data Availability Statement

The datasets presented in this study can be found in online repositories. The names of the repository/repositories and accession number(s) can be found in the article/Supplementary Material.

## Ethics Statement

The studies involving human participants were reviewed and approved by the Institutional Review Board of the University of Nebraska–Lincoln (20160816311EP). The patients/participants provided their written informed consent to participate in this study.

## Author Contributions

AB and DH conceived and directed the project. DH and LP developed the quality-popcorn lines for analysis. NK, LP, MV, QY, PH, JS, DH, and AB contributed to the experimental design. NK and LP optimized *in vitro* digestion protocol. NK performed microbiome collection, grain processing and digestion, starch and protein quantification, microbial fermentation, 16S rRNA sequencing, statistical analysis, gas chromatography, and data visualization. NK, LP, JS, DH, and AB contributed to the writing of the manuscript with input from all other authors. All authors contributed to the article and approved the submitted version.

## Conflict of Interest

The authors declare that the research was conducted in the absence of any commercial or financial relationships that could be construed as a potential conflict of interest.

## Publisher’s Note

All claims expressed in this article are solely those of the authors and do not necessarily represent those of their affiliated organizations, or those of the publisher, the editors and the reviewers. Any product that may be evaluated in this article, or claim that may be made by its manufacturer, is not guaranteed or endorsed by the publisher.

## Supplementary Material

Supplementary Figure 1 Top 50 genera in the baseline microbiomes of the four human subjects based on 16S rRNA sequencing.

Supplementary Figure 2 **(A)** Percentages of total protein in digested popped popcorn flakes as determined by the Bradford assay and compared by the Wilcoxon test (*p* < 0.05). **(B)** Percentages of total starch in digested popped flakes compared by the Wilcoxon test (*p* < 0.05). Values denoted with an “a” had significantly higher starch or protein than those denoted with a “b” by the Wilcoxon test, alpha = 0.05.

Supplementary Figure 3 Relative abundances of genera in all four subjects in response to the non-quality-protein parental hybrid (NQPH) and quality-protein popcorn (QPP) lines. Each bar represents the cumulative data of 8 technical replicates.

Supplementary Figure 4 Alpha diversity described by Inverted Simpson **(A)** and Shannon **(B)** shows patterns in three subjects where lower diversity was observed in microbiomes treated with QPP1B and QPP3B than the other QPP lines.

Supplementary Figure 5 Relative abundance of the *Lachnospiraceae* family is significantly higher in samples treated with QPP than with NQPH in 3 of the 4 human subjects (Mann-Whitney statistic).

Supplementary Figure 6 Relative abundance of genus **(A)** *Coprococcus* in four microbiomes. Relative abundance of bacteria in the **(B)** *Roseburia* and **(C)** *Dorea* genera in two subjects, and **(D)** *Fusobacterium* in one subject post treatment with QPP and NQPH. Comparisons between hybrid pairs tested for significance by the Wilcoxon test (*p* < 0.05).

Supplementary Figure 7 Comparisons (Wilcoxon, *p* < 0.05) between measured butyrate in the microbiomes of four subjects treated with QPP or NQPH.

Supplementary Figure 8 **(A)** Differences in acetate and propionate concentrations in microbiomes of four subjects post treatment with QPP and NQPH lines and comparison (Wilcoxon, *p* < 0.05) between hybrid pairs **(B)** Differences in branched chain fatty acid concentrations in microbiomes of four subjects post treatment with QPP and NQPH lines and comparisons (Wilcoxon, *p* < 0.05).

Supplementary Figure 9 Correlation of ASVs, α-diversity metrics, short chain fatty acid concentrations, pre-digestion lysine and methionine, and post-digestion starch and protein in subject 1 including data from all QPP and NQPH pairs.

Supplementary Figure 10 Inferred pathway enrichment of bacteria in the 4 human subjects; values represent differences in proportions of total sequences in QPP- vs. NQPH-treated microbiomes.

Supplementary Table 1 Amplicon sequence variant (ASV) table generated from 16S rRNA sequencing.

Supplementary Table 2 Genus level table generated from 16S rRNA sequencing.

Supplementary Table 3 MetaCyc pathway enrichment raw data file from PICRUSt.

Supplementary Table 4 ASV and genus level table from 16S rRNA sequencing for FL and lysine fermentation.

Supplementary Table 5 Taxa with significant differential (lysine vs. fructoselysine (FL) vs. media).

Supplementary Data Sheet 1 Taxa with significant differential (quality-protein popcorn, QPP, vs. non-quality-protein parental hybrid, NQPH).

Supplementary Data Sheet 2 Representative sequences of ASVs.

## References

[B1] Akalu G. Taffesse S. Gunaratna N. S. De Groote H. (2010). The effectiveness of quality protein maize in improving the nutritional status of young children in the Ethiopian highlands. *Food Nutr. Bull.* 31 418–430. 10.1177/156482651003100304 20973462

[B2] Akuamoa-Boateng A. (2002). “Quality protein maize: infant feeding trials in Ghana. Ghana health service, Ashanti, Ghana,” in *Experimental Design*, 2nd Edn, eds Cochran W. G. Cox G. M. (New York, NY: John Wiley & Sons, Inc.).

[B3] Alkek Center for Metagenomics and Microbiome Research *ATIMA (Agile Toolkit for Incisive Microbial Analysis*). Houston, TX: Baylor College of Medicine.

[B4] Babu R. Nair S. K. Kumar A. Venkatesh S. Sekhar J. C. Singh N. N. (2005). Two-generation marker-aided backcrossing for rapid conversion of normal maize lines to quality protein maize (QPM). *Theor. Appl. Genet.* 111 888–897. 10.1007/s00122-005-0011-6 16034586

[B5] Bäckhed F. Ding H. Wang T. Hooper L. V. Gou Y. K. Nagy A. (2004). The gut microbiota as an environmental factor that regulates fat storage. *Proc. Natl. Acad. Sci. U.S.A.* 101 15718–15723. 10.1073/pnas.0407076101 15505215 PMC524219

[B6] Bjarnason M. Vasal S. K. (2010). Breeding of quality protein maize (QPM). *Plant Breed. Rev.* 9 181–216. 10.1002/9780470650363.ch7

[B7] Bui T. P. N. Ritari J. Boeren S. De Waard P. Plugge C. M. De Vos W. M. (2015). Production of butyrate from lysine and the Amadori product fructoselysine by a human gut commensal. *Nat. Commun.* 6:10062. 10.1038/ncomms10062 26620920 PMC4697335

[B8] Callahan B. J. McMurdie P. J. Rosen M. J. Han A. W. Johnson A. J. A. Holmes S. P. (2016). DADA2: high-resolution sample inference from illumina amplicon data. *Nat. Methods* 13 581–583. 10.1038/nmeth.3869 27214047 PMC4927377

[B9] Caporaso J. G. Kuczynski J. Stombaugh J. Bittinger K. Bushman F. D. Costello E. K. (2010). QIIME allows analysis of high-throughput community sequencing data. *Nat. Methods* 7 335–336. 10.1038/nmeth.f.303 20383131 PMC3156573

[B10] Chen G. Xie M. Wan P. Chen D. Ye H. Chen L. (2018). Digestion under saliva, simulated gastric and small intestinal conditions and fermentation *in vitro* by human intestinal microbiota of polysaccharides from Fuzhuan brick tea. *Food Chem.* 244 331–339. 10.1016/j.foodchem.2017.10.074 29120790

[B11] Covarrubias-Pazaran G. (2016). Genome-assisted prediction of quantitative traits using the R package sommer. *PLoS One* 11:e0156744. 10.1371/JOURNAL.PONE.0156744 27271781 PMC4894563

[B12] David L. A. Maurice C. F. Carmody R. N. Gootenberg D. B. Button J. E. Wolfe B. E. (2014). Diet rapidly and reproducibly alters the human gut microbiome. *Nature* 505 559–563. 10.1038/nature12820 24336217 PMC3957428

[B13] Douglas G. M. Maffei V. J. Zaneveld J. R. Yurgel S. N. Brown J. R. Taylor C. M. (2020). PICRUSt2 for prediction of metagenome functions. *Nat. Biotechnol.* 38 685–688. 10.1038/s41587-020-0548-6 32483366 PMC7365738

[B14] Gevers H. O. Lake J. K. (1992). “Development of modified opaque-2 maize in South Africa,” in *Quality Protein Maize*, ed. Mertz E. T. (St. Paul, MN: American Association of Cereal Chemists), 49–78.

[B15] Gibbon B. C. Wang X. Larkins B. A. (2003). Altered starch structure is associated with endosperm modification in Quality Protein Maize. *Proc. Natl. Acad. Sci. U.S.A.* 100 15329–15334. 10.1073/pnas.2136854100 14660797 PMC307567

[B16] Graham G. G. Lembcke J. Morales E. (1990). Quality-protein maize as the sole source of dietary protein and fat for rapidly growing young children. *Pediatrics* 85 85–91. 10.1542/peds.85.1.85 2296497

[B17] Hague A. Butt A. J. Paraskeva C. (1996). The role of butyrate in human colonic epithelial cells: an energy source or inducer of differentiation and apoptosis? *Proc. Nutr. Soc.* 55 937–943. 10.1079/PNS19960090 9004335

[B18] He F. (2011). Bradford Protein Assay. *Bio Prot. Bio* 101:e45. 10.21769/bioprotoc.45

[B19] Hellwig M. Henle T. (2014). Baking, ageing, diabetes: a short history of the Maillard reaction. *Angew. Chemie Int. Ed.* 53 10316–10329. 10.1002/anie.201308808 25044982

[B20] Henle T. (2003). AGEs in foods: do they play a role in uremia? *Kidney Int. Suppl.* 84 S145–S147. 10.1046/j.1523-1755.63.s84.16.x 12694332

[B21] Kingston-Smith A. H. Edwards J. E. Huws S. A. Kim E. J. Abberton M. (2010). “Plant-based strategies towards minimising livestock’s long shadow,” in *Proceedings of the Nutrition Society*, (Cambridge: Cambridge University Press), 613–620. 10.1017/S0029665110001953 20682089

[B22] Kostic A. D. Gevers D. Siljander H. Vatanen T. Hyötyläinen T. Hämäläinen A. M. (2015). The dynamics of the human infant gut microbiome in development and in progression toward type 1 diabetes. *Cell Host Microbe* 17 260–273. 10.1016/j.chom.2015.01.001 25662751 PMC4689191

[B23] Kozich J. J. Westcott S. L. Baxter N. T. Highlander S. K. Schloss P. D. (2013). Development of a dual-index sequencing strategy and curation pipeline for analyzing amplicon sequence data on the miseq illumina sequencing platform. *Appl. Environ. Microbiol.* 79 5112–5120. 10.1128/AEM.01043-13 23793624 PMC3753973

[B24] Letunic I. Bork P. (2021). Interactive tree of life (iTOL) v5: an online tool for phylogenetic tree display and annotation. *Nucleic Acids Res.* 49 W293–W296. 10.1093/nar/gkab301 33885785 PMC8265157

[B25] Ley R. E. Lozupone C. A. Hamady M. Knight R. Gordon J. I. (2008). Worlds within worlds: evolution of the vertebrate gut microbiota. *Nat. Rev. Microbiol.* 6 776–788. 10.1038/nrmicro1978 18794915 PMC2664199

[B26] Li J. S. Vasal S. K. (2015). “Maize: quality protein maize,” in *Encyclopedia of Food Grains*, 2nd Edn, eds Wrigley C. W. Corke H. Seetharaman K. Faubion J. (Kidlington: Academic Press), 420–424. 10.1016/B978-0-12-394437-5.00223-0

[B27] Mamatha H. Meena M. Kumar P. C. (2017). Quality protein maize (QPM) as balance nutrition for human diet. *Adv. Plants Agric. Res.* 6 33–35. 10.15406/apar.2017.06.00205

[B28] Manna R. Okello D. K. Imanywoha J. Pixley K. Edema R. (2005). Enhancing introgression of the opaque-2 trait into elite maize lines using simple sequence repeats. *Afr. Crop Sci. J.* 13 215–226. 10.4314/acsj.v13i4

[B29] Mayta-Apaza A. C. Pottgen E. De Bodt J. Papp N. Marasini D. Howard L. (2018). Impact of tart cherries polyphenols on the human gut microbiota and phenolic metabolites *in vitro* and *in vivo*. *J. Nutr. Biochem.* 59 160–172. 10.1016/j.jnutbio.2018.04.001 30055451

[B30] Meehan C. J. Beiko R. G. (2014). A phylogenomic view of ecological specialization in the lachnospiraceae, a family of digestive tract-associated bacteria. *Genome Biol. Evol.* 6 703–713. 10.1093/gbe/evu050 24625961 PMC3971600

[B31] Mertz E. T. Bates L. S. Nelson O. E. (1964). Mutant gene that changes protein composition and increases lysine content of maize endosperm. *Science* 145 279–280. 10.1126/science.145.3629.279 14171571

[B32] Morton K. J. Jia S. Zhang C. Holding D. R. (2016). Proteomic profiling of maize opaque endosperm mutants reveals selective accumulation of lysine-enriched proteins. *J. Exp. Bot.* 67 1381–1396. 10.1093/jxb/erv532 26712829 PMC4762381

[B33] Namiki M. Hayashi T. (1983). “A new mechanism of the Maillard Reaction Involving Sugar Fragmentation and Free Radical Formation,” in *The Maillard Reaction in Foods and Nutrition*, eds Waller G. R. Feather M. S. (Washington, D.C: ACS Publication), 21–46. 10.1021/bk-1983-0215.ch002

[B34] Nuss E. T. Tanumihardjo S. A. (2011). Quality protein maize for Africa: closing the protein inadequacy gap in vulnerable populations. *Adv. Nutr.* 2 217–224. 10.3945/an.110.000182 22332054 PMC3090170

[B35] Nyangale E. P. Mottram D. S. Gibson G. R. (2012). Gut microbial activity, implications for health and disease: the potential role of metabolite analysis. *J. Proteome Res.* 11 5573–5585. 10.1021/PR300637D 23116228

[B36] Parsons L. Ren Y. Yobi A. Angelovici R. Rodriguez O. Holding D. R. (2021a). Final selection of quality protein popcorn hybrids. *Front. Plant Sci.* 12:658456. 10.3389/fpls.2021.658456 33841483 PMC8025670

[B37] Parsons L. Ren Y. Yobi A. Hurst P. Angelovici R. Rodriguez O. (2020). Production and selection of quality protein popcorn hybrids using a novel ranking system and combining ability estimates. *Front. Plant Sci.* 11:698. 10.3389/fpls.2020.00698 32655587 PMC7325744

[B38] Parsons L. Rodriguez O. Holding D. R. (2021b). Improved taste and texture in novel popcorn varieties compared to conventional lines. *J. Sens. Stud.* 36:e12687. 10.1111/joss.12687

[B39] Quast C. Pruesse E. Yilmaz P. Gerken J. Schweer T. Yarza P. (2013). The SILVA ribosomal RNA gene database project: improved data processing and web-based tools. *Nucleic Acids Res.* 41:D590. 10.1093/nar/gks1219 23193283 PMC3531112

[B40] R Core Team. (2020). *R: A Language and Environment for Statistical Computing.* Vienna: R Foundation for Statistical Computing.

[B41] Ren Y. Yobi A. Marshall L. Angelovici R. Rodriguez O. Holding D. R. (2018). Generation and evaluation of modified opaque-2 popcorn suggests a route to quality protein popcorn. *Front. Plant Sci.* 871:1803. 10.3389/fpls.2018.01803 30574157 PMC6291453

[B42] Salamini F. Borghi B. Lorenzoni C. (1970). The effect of the opaque-2 gene on yield in maize. *Euphytica* 19 531–538. 10.1007/BF01902928

[B43] Shang H. Sun J. Chen Y. Q. (2016). *Clostridium butyricum* CGMCC0313.1 modulates lipid profile, insulin resistance and colon homeostasis in obese Mice. *PLoS One* 11:e0154373. 10.1371/JOURNAL.PONE.0154373 27123997 PMC4849746

[B44] Sofi P. A. Wani S. A. Rather A. G. Wani S. H. (2009). Quality protein maize (QPM): genetic manipulation for the nutritional fortification of maize. *J. Plant Breed. Crop Sci.* 1 244–253.

[B45] Stams A. J. M. Van Dijk J. B. Dijkema C. Plugge C. M. (1993). Growth of syntrophic propionate-oxidizing bacteria with fumarate in the absence of methanogenic bacteria. *Appl. Environ. Microbiol.* 59 1114–1119. 10.1128/aem.59.4.1114-1119.1993 16348912 PMC202247

[B46] Swendseid M. E. (1981). Essential Amino Acid Requirements: A Review. FAO/WHO/UNU Expert Consultation on Energy and Protein Requirements. Available online at: http://www.fao.org/3/m2772e/m2772e00.htm (accessed September 9 2021).

[B47] Tang W. H. W. Wang Z. Levison B. S. Koeth R. A. Britt E. B. Fu X. (2013). Intestinal microbial metabolism of phosphatidylcholine and cardiovascular risk. *N. Engl. J. Med.* 368 1575–1584. 10.1056/nejmoa1109400 23614584 PMC3701945

[B48] Thomsen M. C. F. Hasman H. Westh H. Kaya H. Lund O. (2017). RUCS: rapid identification of PCR primers for unique core sequences. *Bioinformatics* 33 3917–3921. 10.1093/bioinformatics/btx526 28968748 PMC5860091

[B49] Tuncil Y. E. Thakkar R. D. Arioglu-Tuncil S. Hamaker B. R. Lindemann S. R. (2018). Fecal microbiota responses to bran particles are specific to cereal type and *in vitro* digestion methods that mimic upper gastrointestinal tract passage. *J. Agric. Food Chem.* 66 12580–12593. 10.1021/acs.jafc.8b03469 30406656

[B50] Turnbaugh P. J. Ridaura V. K. Faith J. J. Rey F. E. Knight R. Gordon J. I. (2009). The effect of diet on the human gut microbiome: a metagenomic analysis in humanized gnotobiotic mice. *Sci. Transl. Med.* 1:6ra14. 10.1126/scitranslmed.3000322 20368178 PMC2894525

[B51] Vasal S. K. (2000). The quality protein maize story. *Food Nutr. Bull.* 21 445–450. 10.1177/156482650002100420

[B52] Vasal S. K. Villegas E. Bjarnason M. Gelaw B. Goertz P. (1980). “Genetic modifiers and breeding strategies in developing hard endosperm opaque-2 materials,” in *Proceedings of the Improvement of Quality Traits of Maize for Grain and Silage Use*, 37–73. Available online at: https://www.cabdirect.org/cabdirect/abstract/19811605259 (accessed March 9, 2022).

[B53] Velázquez O. C. Lederer H. M. Rombeau J. L. (1997). Butyrate and the colonocyte. *Adv. Exp. Med. Biol.* 427 123–134. 10.1007/978-1-4615-5967-2_14 9361838

[B54] Vital M. Howe A. C. Tiedje J. M. (2014). Revealing the bacterial butyrate synthesis pathways by analyzing (meta)genomic data. *mBio* 5:e00889. 10.1128/mBio.00889-14 24757212 PMC3994512

[B55] Wang H.-B. Wang P.-Y. Wang X. Wan Y.-L. Liu Y.-C. (2012). Butyrate enhances intestinal epithelial barrier function *via* up-regulation of tight junction protein Claudin-1 transcription. *Dig. Dis. Sci.* 57 3126–3135. 10.1007/S10620-012-2259-4 22684624

[B56] Willett W. Rockström J. Loken B. Springmann M. Lang T. Vermeulen S. (2019). Food in the anthropocene: the EAT–Lancet commission on healthy diets from sustainable food systems. *Lancet* 393 447–492. 10.1016/S0140-6736(18)31788-4 30660336

[B57] World Health Organization [WHO] (2003). Diet, nutrition and the prevention of chronic diseases. *World Health Organ. Tech. Rep. Ser.* 916 i–viii, 1–149. 12768890

[B58] Wu G. D. Chen J. Hoffmann C. Bittinger K. Chen Y. Y. Keilbaugh S. A. (2011). Linking long-term dietary patterns with gut microbial enterotypes. *Science* 334 105–108. 10.1126/science.1208344 21885731 PMC3368382

[B59] Yang J. Rose D. J. (2014). Long-term dietary pattern of fecal donor correlates with butyrate production and markers of protein fermentation during *in vitro* fecal fermentation. *Nutr. Res.* 34 749–759. 10.1016/j.nutres.2014.08.006 25218569

[B60] Yang J. Martínez I. Walter J. Keshavarzian A. Rose D. J. (2013). Invitro characterization of the impact of selected dietary fibers on fecal microbiota composition and short chain fatty acid production. *Anaerobe* 23 74–81. 10.1016/j.anaerobe.2013.06.012 23831725

[B61] Yang Q. Van Haute M. Korth N. Sattler S. Rose D. Juritsch A. (2022). Near isogenic lines (NIL) of sorghum carrying wild type or waxy alleles of the granule-bound starch synthase (GBSS) gene have distinct effects on human gut microbiome phenotypes and host physiological characteristics. *Res. Squ.* [Preprint]. 10.21203/RS.3.RS-1405055/V1

[B62] Ye J. Coulouris G. Zaretskaya I. Cutcutache I. Rozen S. Madden T. L. (2012). Primer-BLAST: a tool to design target-specific primers for polymerase chain reaction. *BMC Bioinformatics* 13:134. 10.1186/1471-2105-13-134 22708584 PMC3412702

[B63] Zhou J. Hegsted M. McCutcheon K. L. Keenan M. J. Xi A. Raggio A. M. (2006). Peptide YY and proglucagon mRNA expression patterns and regulation in the gut. *Obesity* 14 683–689. 10.1038/oby.2006.77 16741270

[B64] Zhu L. Clayton J. B. Suhr Van Haute M. J. Yang Q. Hassenstab H. R. Mustoe A. C. (2020). Sex bias in gut microbiome transmission in newly paired marmosets (*Callithrix jacchus*). *mSystems* 5:e00910-19. 10.1128/mSystems.00910-19 32209720 PMC7093826

