# Supplementary figures and images for: The Unique Seed Protein Composition of Quality Protein Popcorn Promotes Growth of Beneficial Bacteria From the Human Gut Microbiome
Source: Front Microbiol. 2022 Jul 14;13:921456. doi: 10.3389/fmicb.2022.921456 (PMC9330393; doi:10.3389/fmicb.2022.921456)

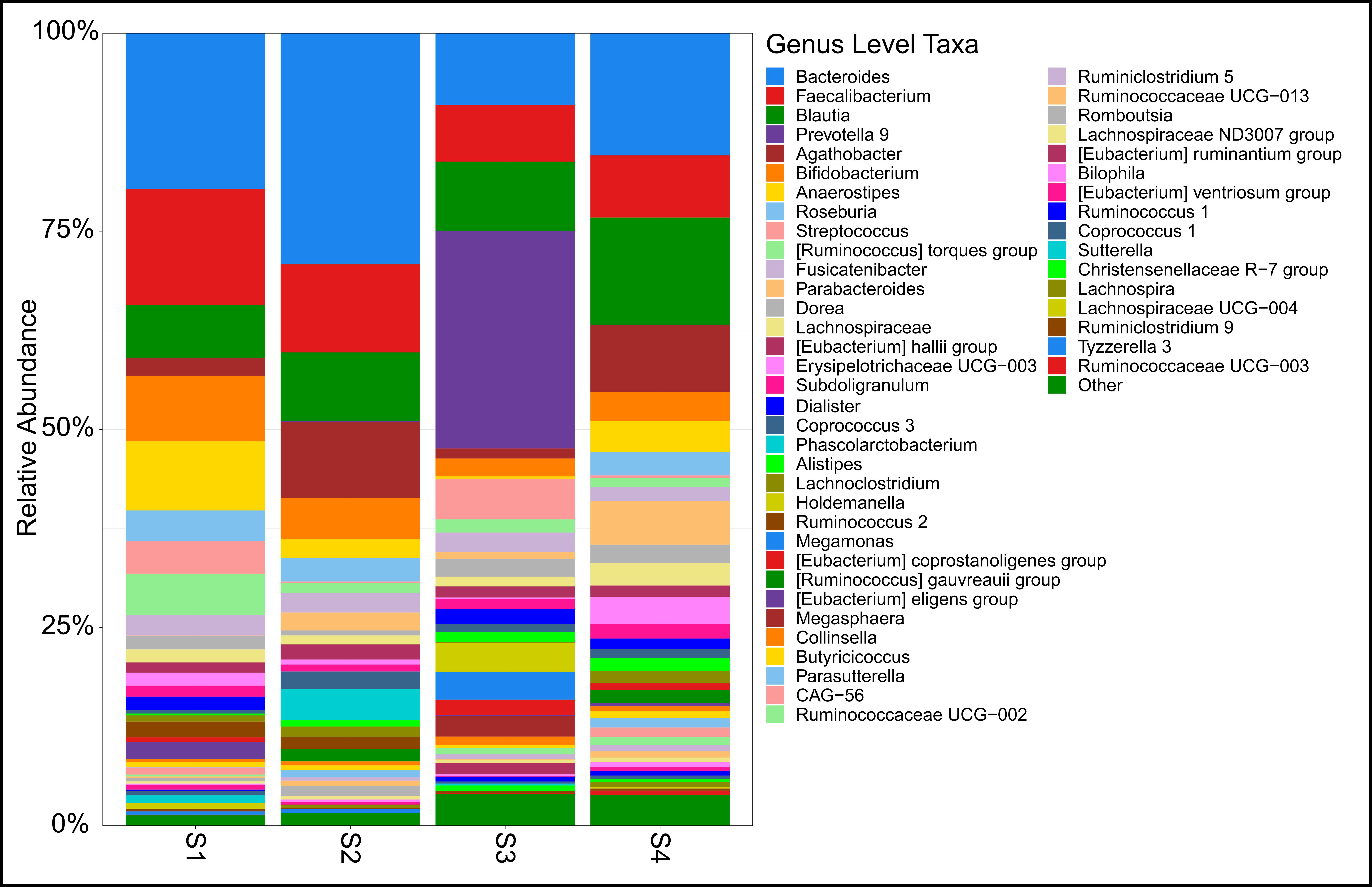

Supplement: Supplementary Figure 1 — Top 50 genera in the baseline microbiomes of the four human subjects based on 16S rRNA sequencing. [file Image_1.JPEG]

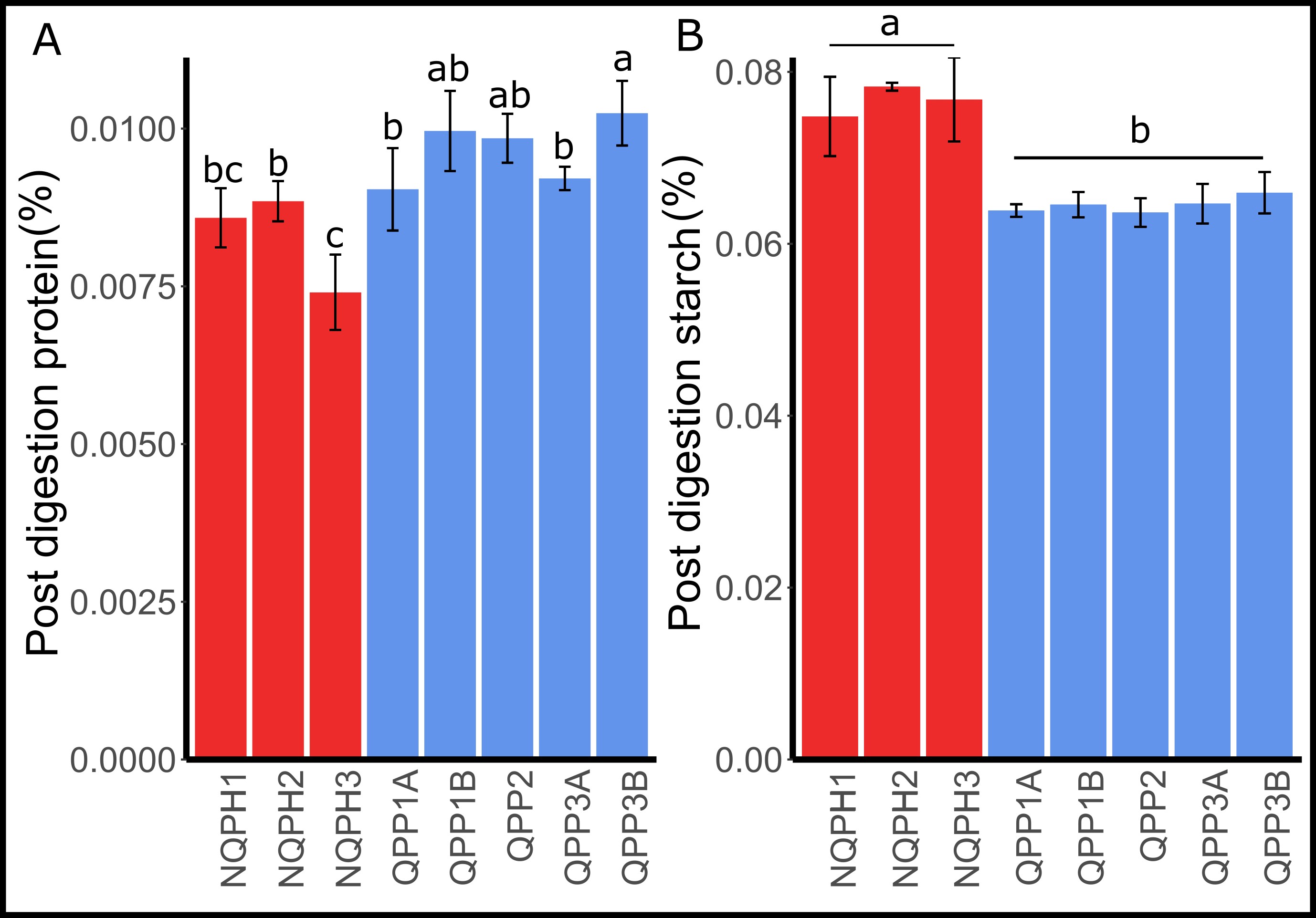

Supplement: Supplementary Figure 2 — (A) Percentages of total protein in digested popped popcorn flakes as determined by the Bradford assay and compared by the Wilcoxon test (p < 0.05). (B) Percentages of total starch in digested popped flakes compared by the Wilcoxon test (p < 0.05). Values denoted with an “a” had significantly higher starch or protein than those denoted with a “b” by the Wilcoxon test, alpha = 0.05. [file Image_2.jpg]

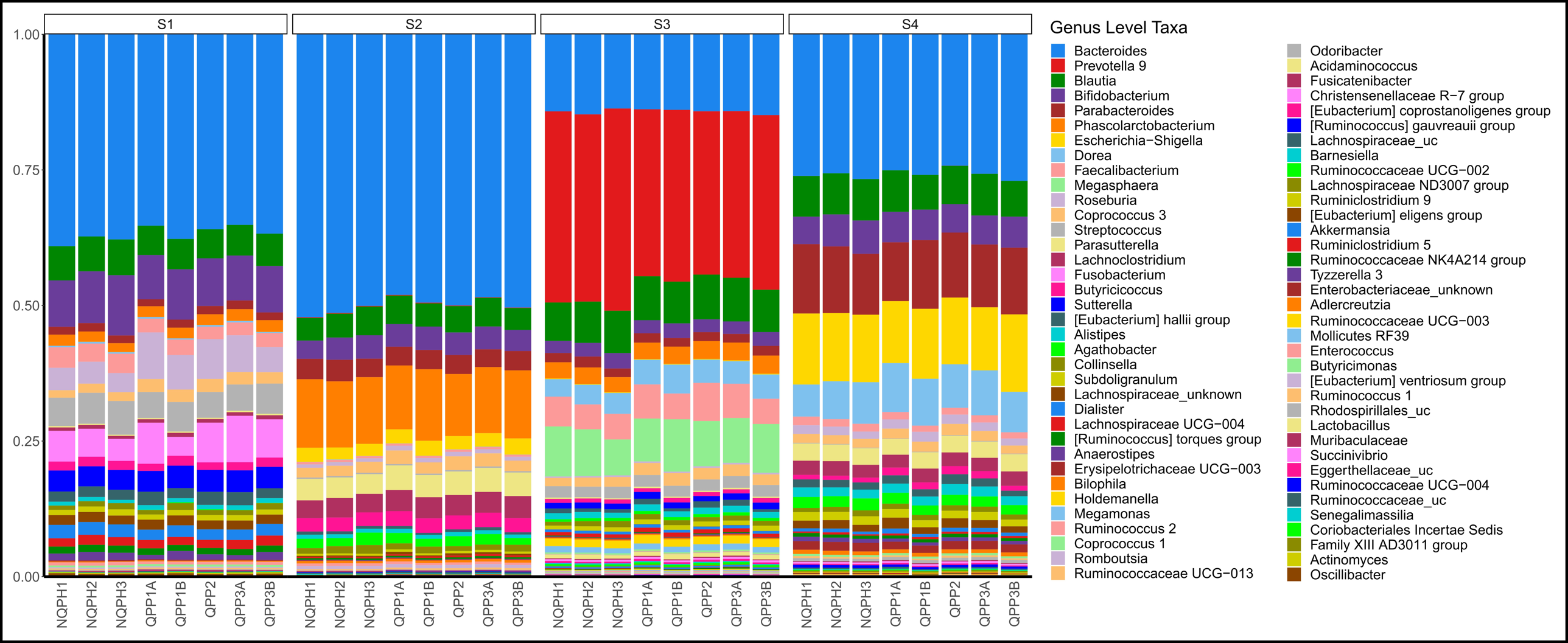

Supplement: Supplementary Figure 3 — Relative abundances of genera in all four subjects in response to the non-quality-protein parental hybrid (NQPH) and quality-protein popcorn (QPP) lines. Each bar represents the cumulative data of 8 technical replicates. [file Image_3.JPEG]

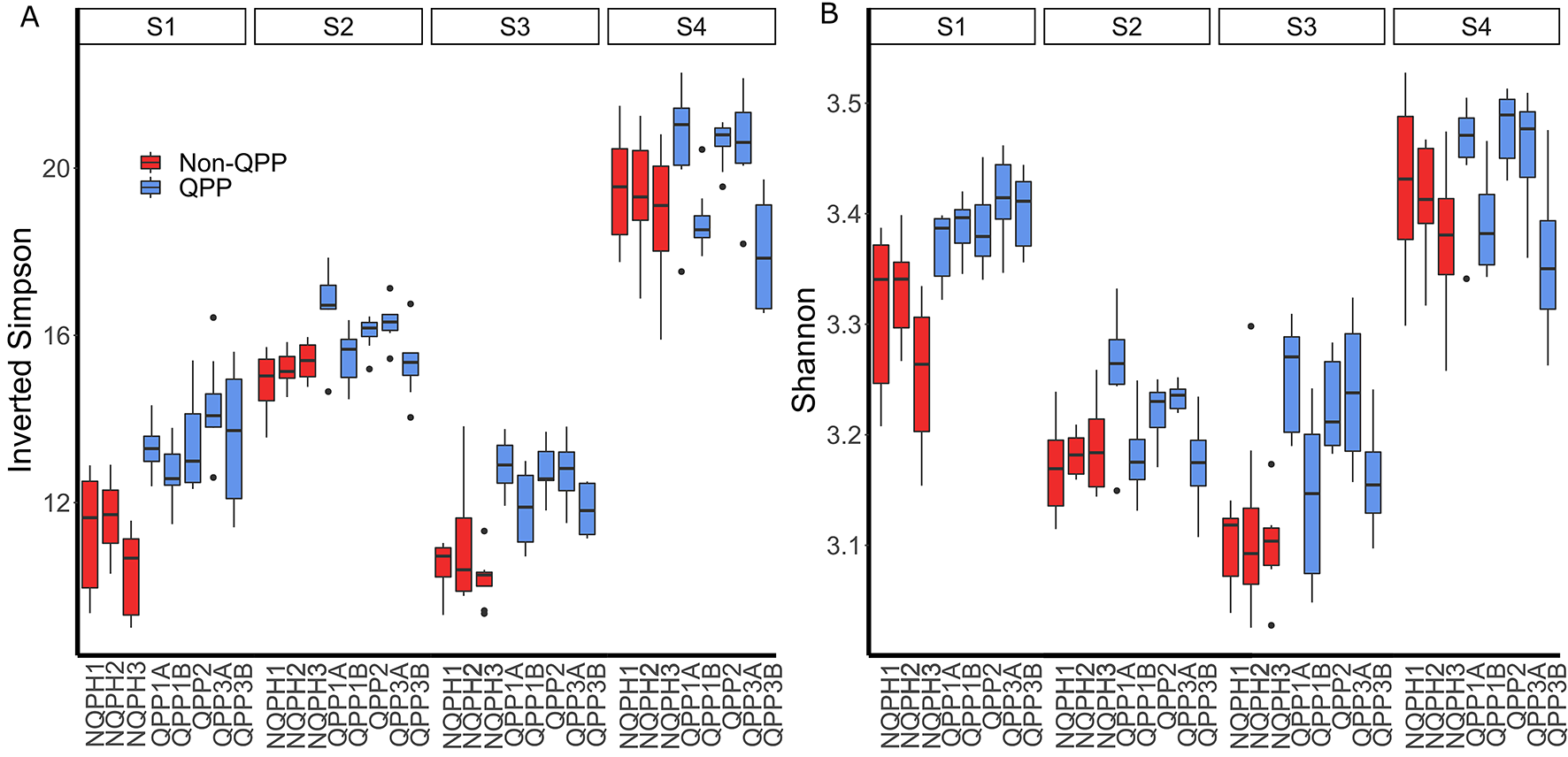

Supplement: Supplementary Figure 4 — Alpha diversity described by Inverted Simpson (A) and Shannon (B) shows patterns in three subjects where lower diversity was observed in microbiomes treated with QPP1B and QPP3B than the other QPP lines. [file Image_4.TIFF]

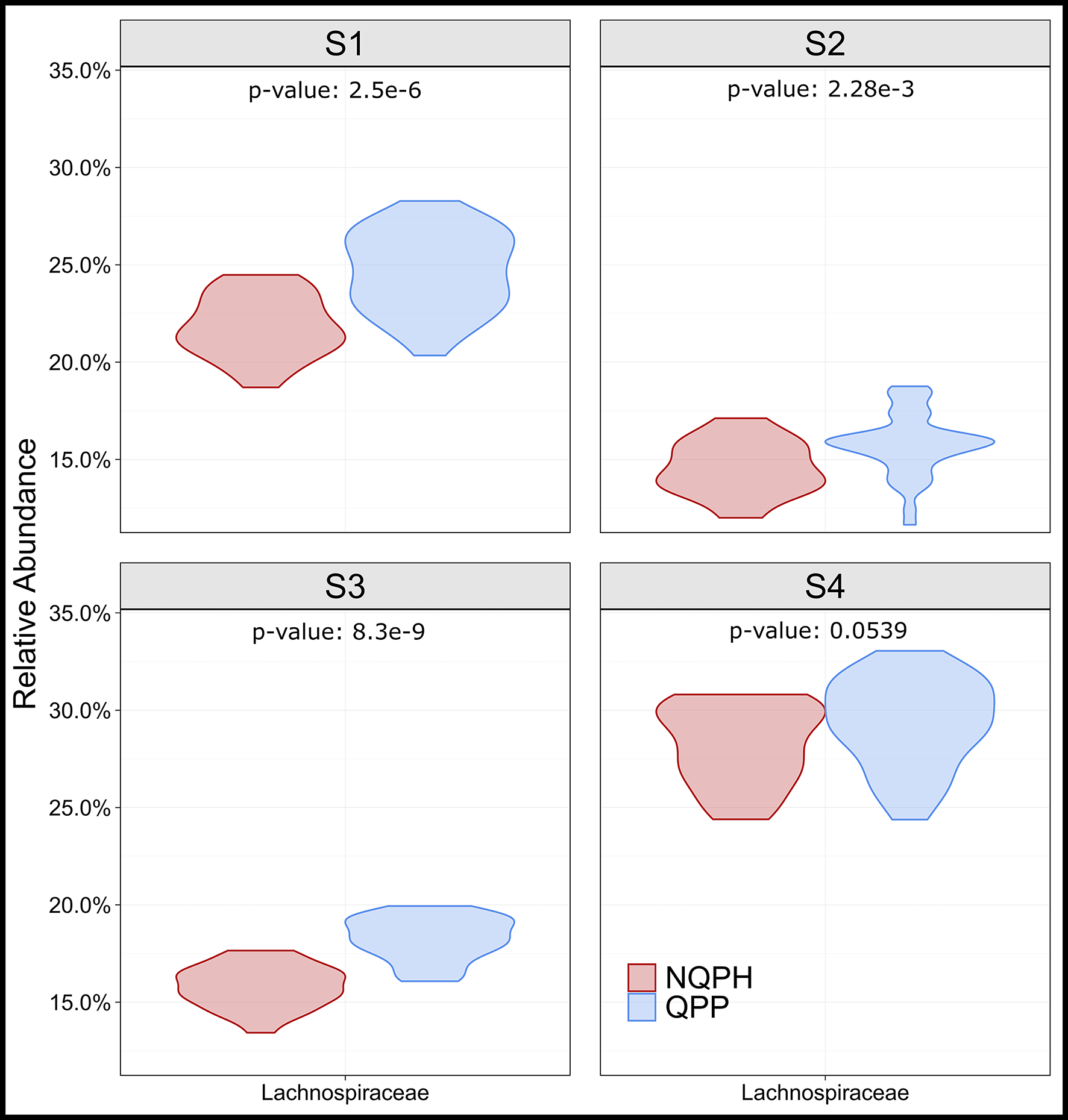

Supplement: Supplementary Figure 5 — Relative abundance of the Lachnospiraceae family is significantly higher in samples treated with QPP than with NQPH in 3 of the 4 human subjects (Mann-Whitney statistic). [file Image_5.TIFF]

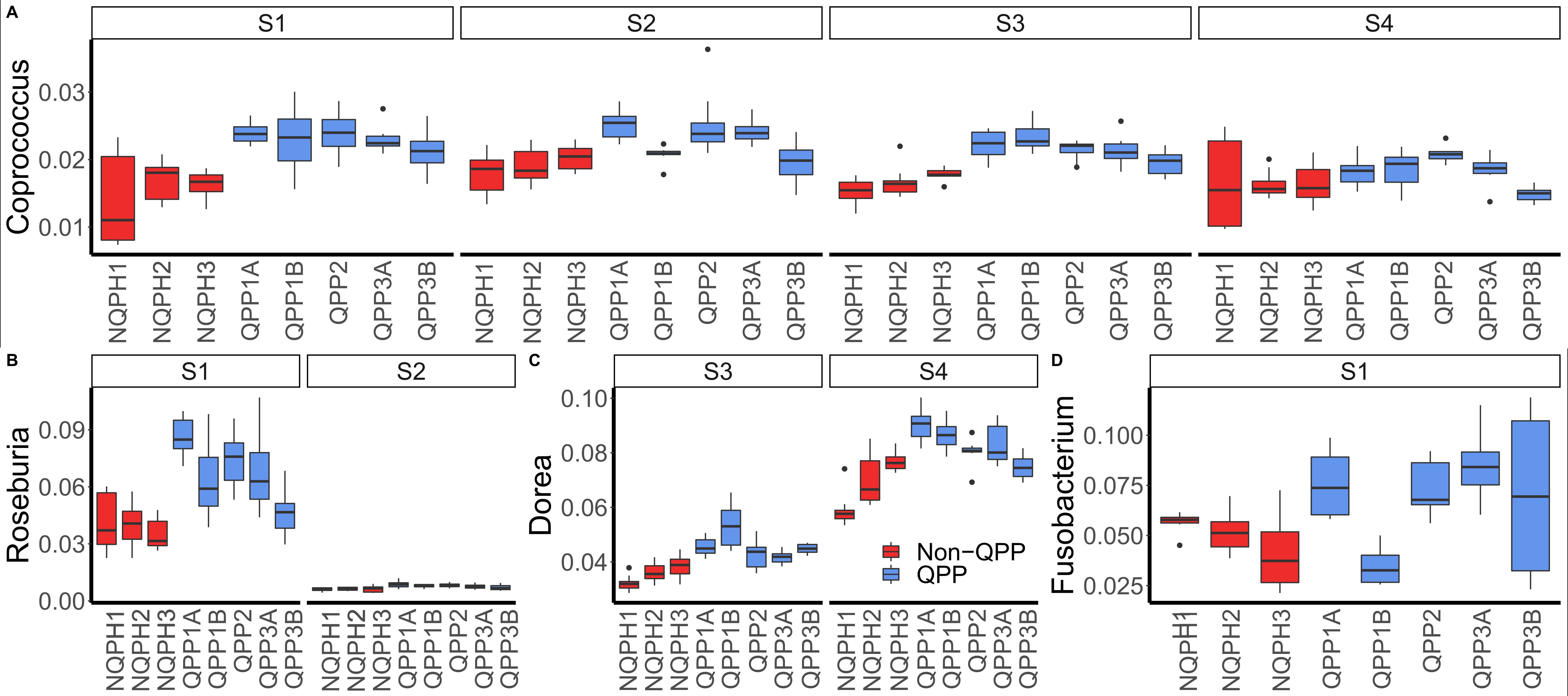

Supplement: Supplementary Figure 6 — Relative abundance of genus (A) Coprococcus in four microbiomes. Relative abundance of bacteria in the (B) Roseburia and (C) Dorea genera in two subjects, and (D) Fusobacterium in one subject post treatment with QPP and NQPH. Comparisons between hybrid pairs tested for significance by the Wilcoxon test (p < 0.05). [file Image_6.JPEG]

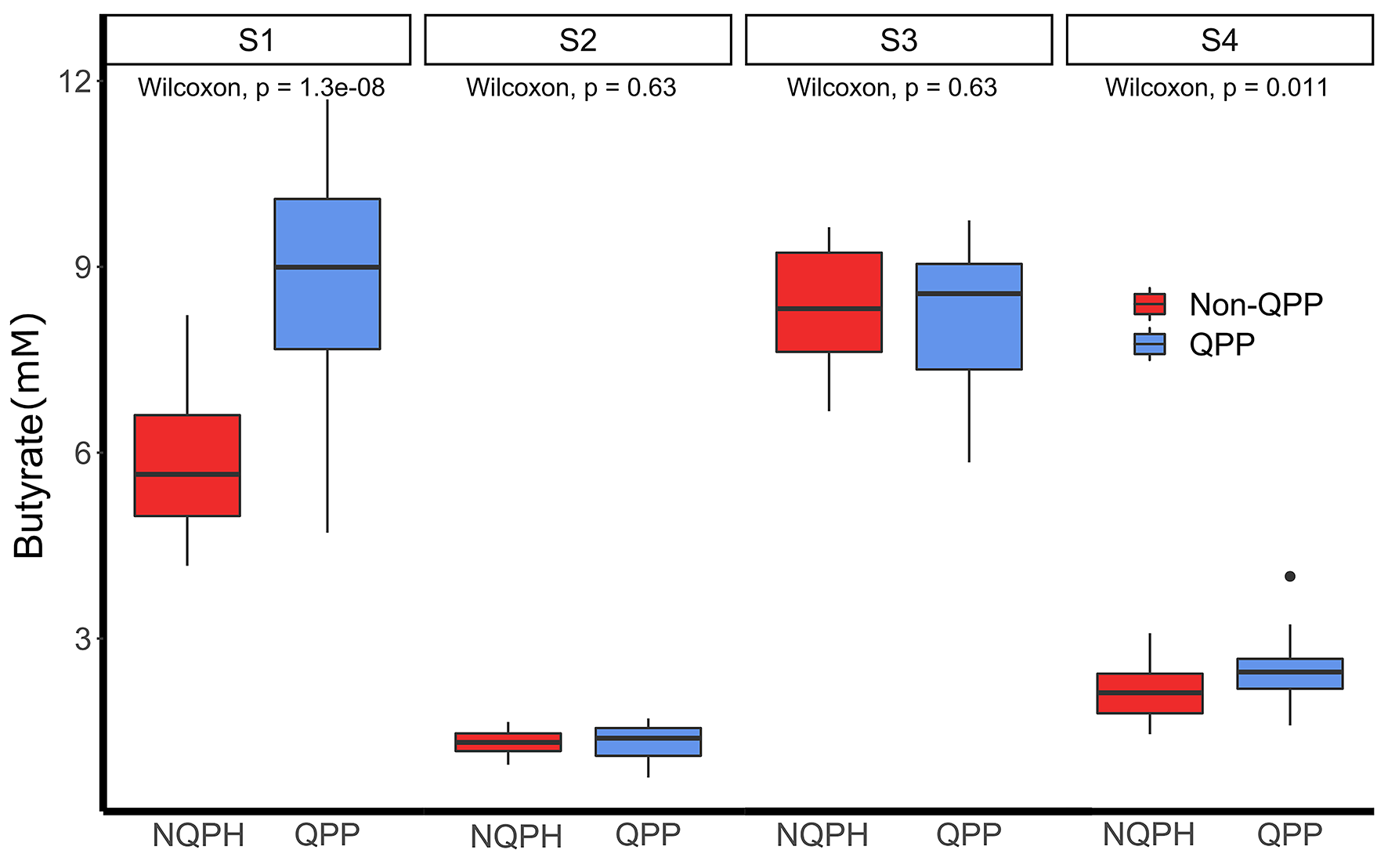

Supplement: Supplementary Figure 7 — Comparisons (Wilcoxon, p < 0.05) between measured butyrate in the microbiomes of four subjects treated with QPP or NQPH. [file Image_7.TIFF]

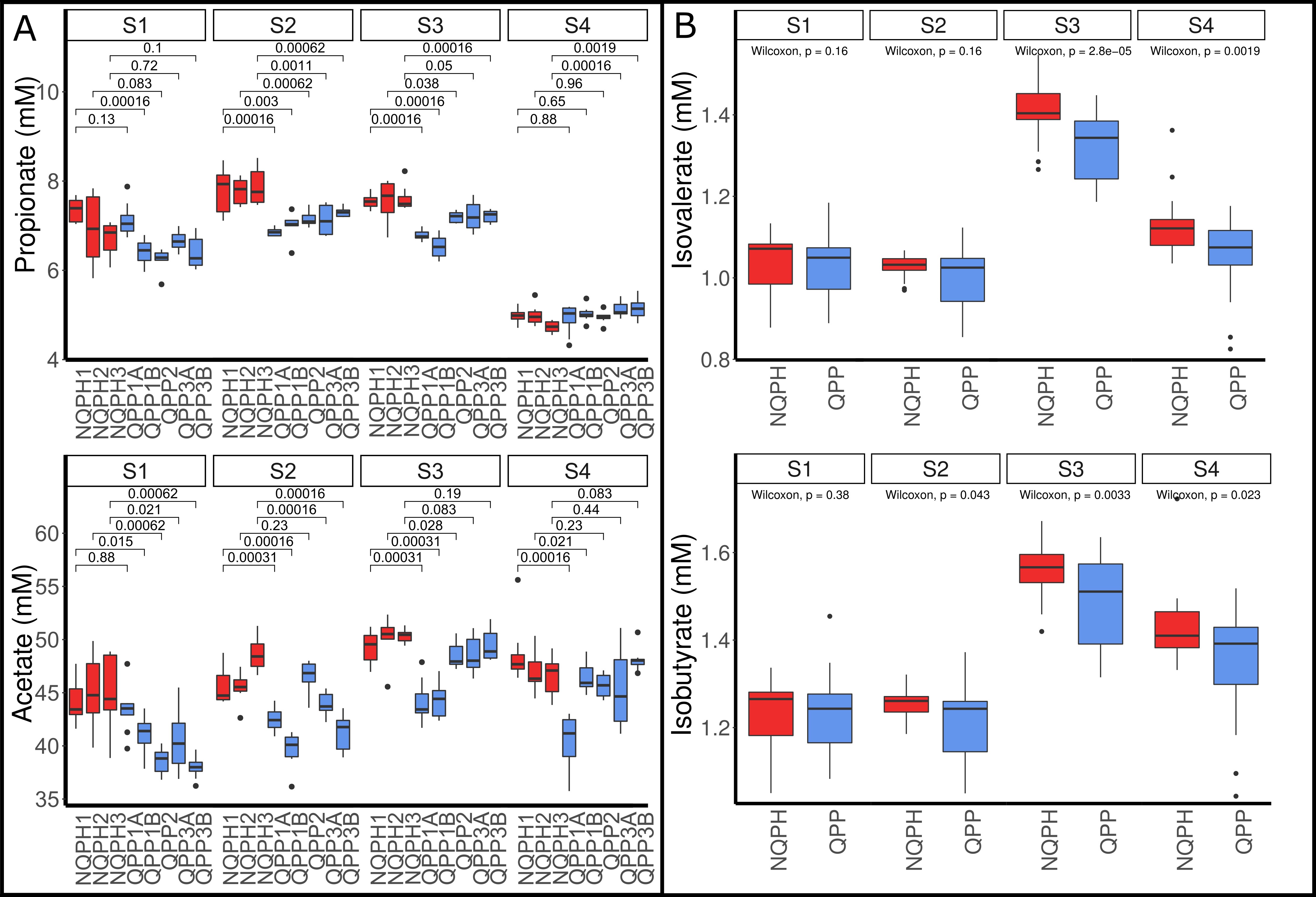

Supplement: Supplementary Figure 8 — (A) Differences in acetate and propionate concentrations in microbiomes of four subjects post treatment with QPP and NQPH lines and comparison (Wilcoxon, p < 0.05) between hybrid pairs (B) Differences in branched chain fatty acid concentrations in microbiomes of four subjects post treatment with QPP and NQPH lines and comparisons (Wilcoxon, p < 0.05). [file Image_8.jpg]

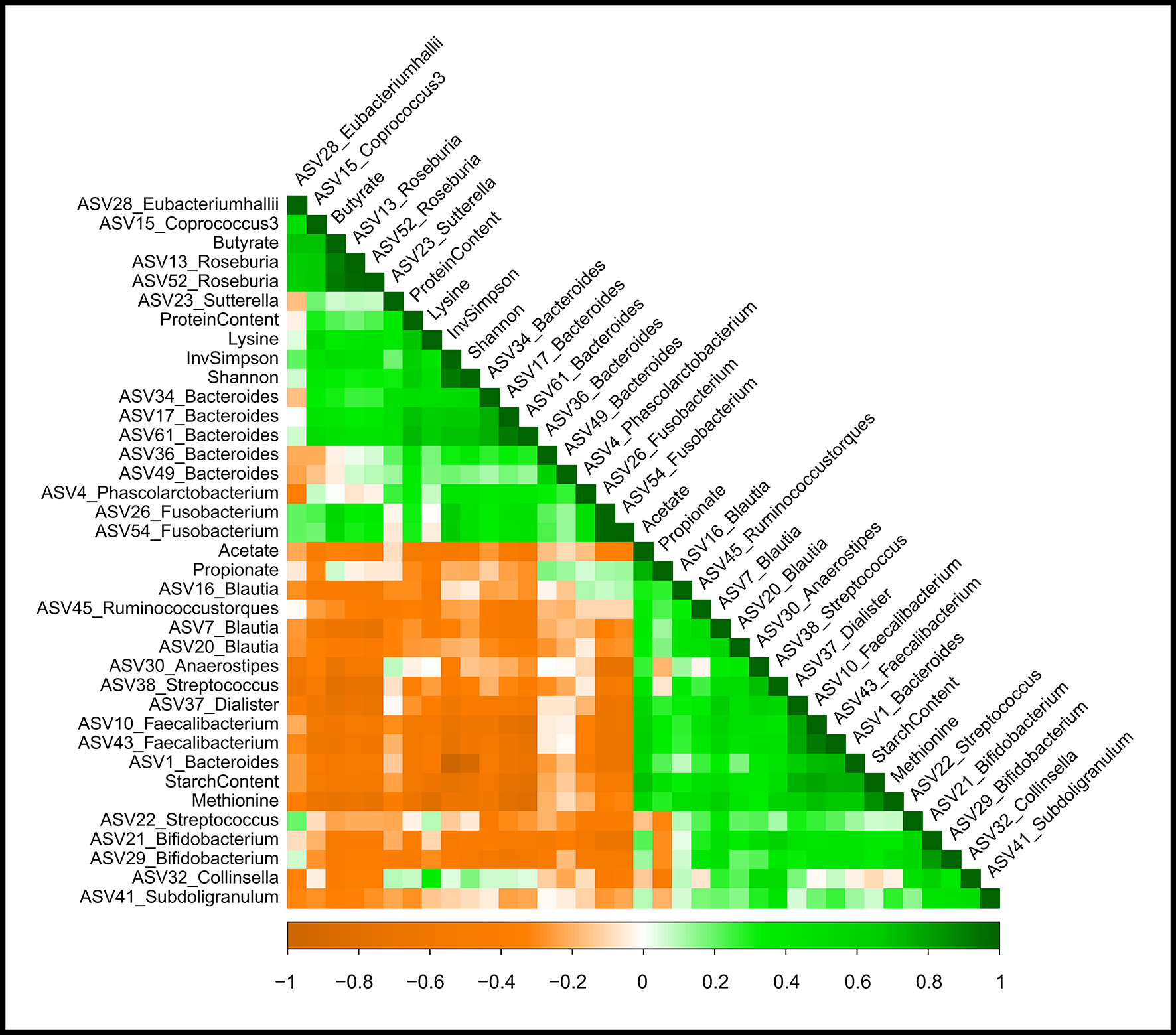

Supplement: Supplementary Figure 9 — Correlation of ASVs, α-diversity metrics, short chain fatty acid concentrations, pre-digestion lysine and methionine, and post-digestion starch and protein in subject 1 including data from all QPP and NQPH pairs. [file Image_9.TIFF]

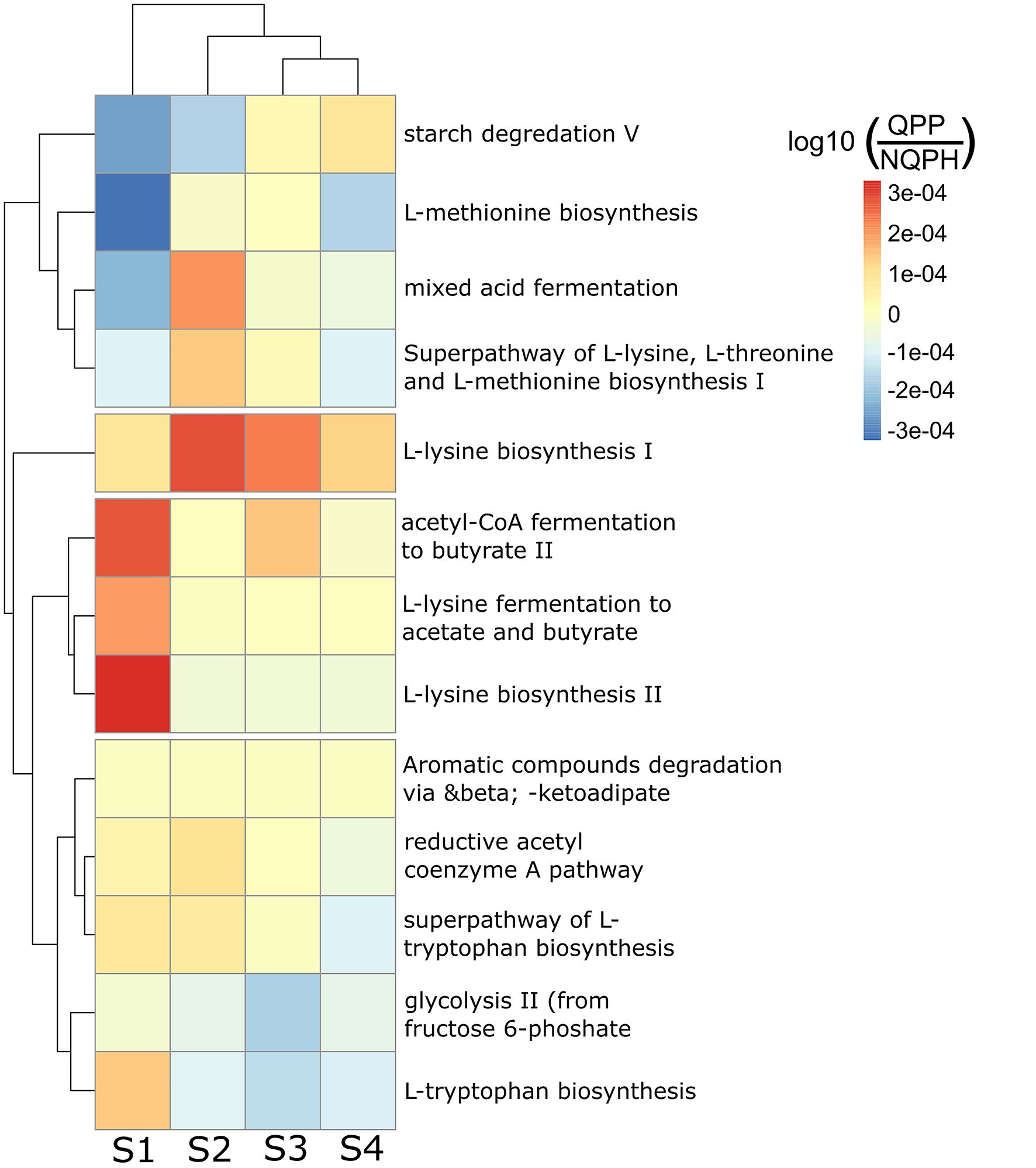

Supplement: Supplementary Figure 10 — Inferred pathway enrichment of bacteria in the 4 human subjects; values represent differences in proportions of total sequences in QPP- vs. NQPH-treated microbiomes. [file Image_10.TIFF]
